# Blood pressure control after solid organ transplantation: opportunities for optimizing care

**DOI:** 10.3389/ti.2026.16565

**Published:** 2026-07-30

**Authors:** A. M. Posthumus, T. J. Knobbe, D. Kremer, M. F. Eisenga, J. S. F. Sanders, S. P. Berger, C. Smit, F. Klont, C. T. Gan, E. A. M. Verschuuren, K. Damman, M. H. de Borst, H. Blokzijl, S. J. L. Bakker, V. E. de Meijer

**Affiliations:** 1 Department of Internal Medicine, University Medical Center Groningen, University of Groningen, Groningen, Netherlands; 2 Department of Clinical Pharmacy and Pharmacology, University Medical Center Groningen, University of Groningen, Groningen, Netherlands; 3 Unit of Pharmacotherapy, Epidemiology and Economics, Groningen Research Institute of Pharmacy, University of Groningen, Groningen, Netherlands; 4 Department of Pulmonary Diseases and Tuberculosis, University Medical Center Groningen, University of Groningen, Groningen, Netherlands; 5 Department of Cardiology, University Medical Center Groningen, University of Groningen, Groningen, Netherlands; 6 Department of Gastroenterology and Hepatology, University Medical Center Groningen, University of Groningen, Groningen, Netherlands; 7 Department of Surgery, University Medical Center Groningen, University of Groningen, Groningen, Netherlands

**Keywords:** blood pressure management, hypertension, solid organ transplantation, suboptimal treatment, uncontrolled hypertension

## Abstract

Hypertension affects 50%–90% of solid organ transplant recipients (SOTR) and is a major driver of cardiovascular disease. Nevertheless, hypertension control has received little attention in this population. We assessed blood pressure control 1 year after heart, liver, lung or kidney transplantation using cross-sectional data from 1112 SOTR (39% female, mean age 57 ± 13 years) from the TransplantLines biobank and cohort study. Suboptimal control was defined as systolic blood pressure >130 mmHg or diastolic blood pressure >80 mmHg. Overall, 997 (90%) SOTR had hypertension. Suboptimal control occurred in 721 (72%), including 146 (20%) who received no antihypertensive treatment despite elevated blood pressure. Rates of suboptimal control were consistently high across organ types (71%–84%). Older age (OR = 1.03; 95%CI1.01–1.04), diabetes (OR = 1.99; 95%CI1.18–3.36), and higher cholesterol (OR = 1.23; 95%CI1.01–1.51) were independently associated with suboptimal control. Among treated SOTR, recipients were older (OR = 1.03; 95%CI1.01–1.05), more often male (OR = 1.55; 95%CI1.03–2.34), and had more prior cardiovascular events (OR = 2.06; 95%CI1.14–3.95). In sensitivity analyses using alternative blood pressure thresholds, suboptimal control remained common, affecting 40% of hypertensive SOTR using a ≤140/90 mmHg threshold. In conclusion, nearly three out of four hypertensive SOTR have suboptimal blood pressure control at 1 year after transplantation with a ≤130/80 mmHg threshold, highlighting a substantial care gap in post-transplant management.

## Graphical Abstract

**Figure:**
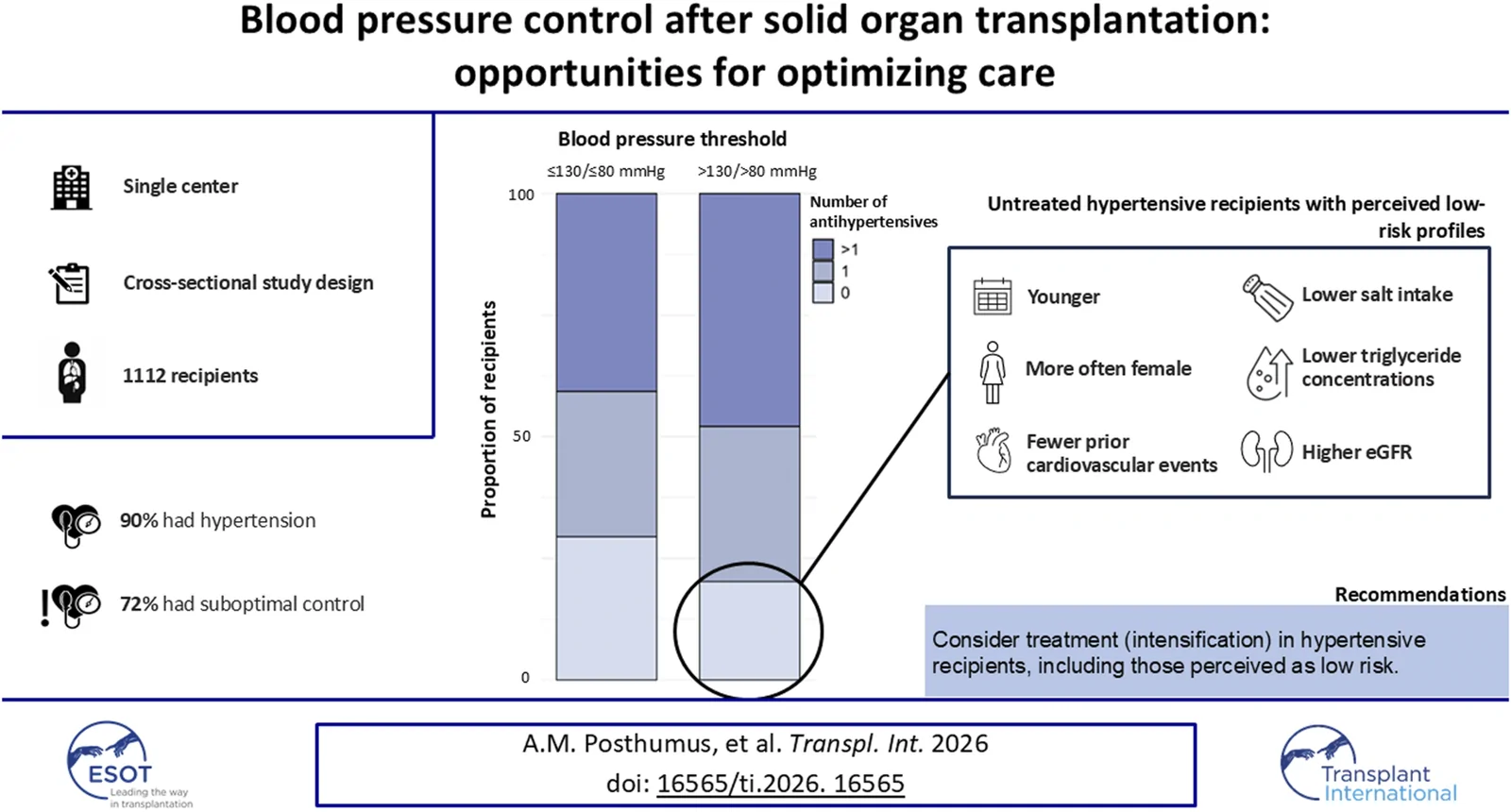

## Introduction

Solid organ transplantation (SOT) is a lifesaving procedure, which not only extends the life-span of recipients with organ failure, but also improves their health-related quality of life [1]. However, even after successful SOT there is still an increased risk for cardiovascular disease (CVD) [2]. CVD is an acknowledged leading cause of morbidity and mortality in SOT recipients [2–4]. Risk for CVD varies between different SOT programs (heart, liver, lung, kidney) [5], but is higher compared to the general population [6–8]. Various factors contribute to CVD, particularly hypertension [2]. In SOT recipients, side effects of immunosuppressive medication, including corticosteroids and calcineurin inhibitors, endothelial dysfunction, and kidney dysfunction, contribute to the occurrence of hypertension [9–11]. Consequently, hypertension is highly prevalent among SOT recipients, affecting 50%–90% of recipients [12–16].

No clear consensus exists regarding blood pressure targets in SOT recipients. Current guidelines recommend systolic blood pressure (SBP) targets ranging from <120 to <140 mmHg and diastolic blood pressure (DBP) targets ranging from <80 to <90 mmHg [17], while most consensus supporting a target of ≤130/80 mmHg [18–22].

In liver transplant recipients, optimal blood pressure control was associated with a 45% reduction in mortality and 38% reduction in cardiovascular events [23]. Additionally, hypertension can contribute to decline of kidney function, which is common among SOT recipients [24].

Globally, approximately half of the general population with hypertension is unaware of their diagnosis, and 80% of those diagnosed have suboptimal control according to the World Health Organization [25]. To the best of our knowledge, this topic has received limited attention in the SOT population. While blood pressure control is a major objective in transplant recipients, evidence on the adequacy of achieved blood pressure control remains scarce. This may in part be explained by the earlier focus on short-term outcomes after transplantation. As survival after SOT continues to improve, long-term outcomes are becoming increasingly important, and cardiovascular risk management deserves greater attention. We therefore aimed to investigate hypertension control in SOT recipients at 1-year after transplantation. Additionally, we determined whether hypertension control differed across SOT programs (heart, liver, lung, kidney) in our center.

## Materials and methods

### Patient population and study design

Cross-sectional data were used from the ongoing and prospective TransplantLines Biobank and Cohort Study (ClinicalTrials.gov identifier: NCT03272841; METc 2014/077). This study collected data from all SOT recipients and donors (aged ≥18 years) with a written informed consent, starting from June 2015 at the University Medical Centre Groningen (UMCG, Netherlands). TransplantLines adheres with the UMCG Biobank Regulation, WMA Declaration of Helsinki and the Declaration of Istanbul [26, 27].

In the current study, we included adult (aged ≥18 years at inclusion) SOT recipients at 1-year after SOT (liver, lung, heart and kidney recipients). Assessments were performed during the standardized 1-year post-transplant visit, which occurred within a predefined window of approximately 9–16 months after transplantation. Data between June 2015 and December 2024 were used. If recipients had multiple transplant trajectories included in TransplantLines, data from the first transplant trajectory were used if the first transplant occurred at least 1 year before the second transplant, and the allograft did not fail. We excluded recipients who had a combined SOT. This study was performed in accordance with the STrengthening the Reporting of OBservational studies in Epidemiology (STROBE) statement (Supplementary Table S1).

### Covariables

Before and including 2021, clinical data and data on medication use (verified with the patient) were collected during TransplantLines study visits. SBP and DBP, were measured in seated position after 15 min of rest, with four consecutive seated measurements. Waist circumference was measured at least twice. Mean value was used if multiple measurements were available [28]. After 2021, study visits were discontinued, and data were extracted from the electronic patient dossier (EPD), with blood pressure values including both patient-reported and physician-recorded measurements, which ranged from single measurements or averages of repeated measurements. Laboratory values were obtained through standard laboratory measurements. For the calculation of eGFR, the 2009 CKD-EPI formula was used [29]. Metabolic syndrome was defined according to the criteria of the International Diabetes Federation [30]. Instead of fasting glucose, HbA1c was used with a threshold of 53 mmol/mol, as not all patients were able to fast at the time of blood sampling due to long travel times to our transplant center. Cardiovascular events (CVE) were defined as the occurrence of myocardial infarction, heart failure, peripheral vascular disease, cerebrovascular accident (CVA), or transient ischemic attack (TIA). These events were obtained from EPD using ICD-10 codes. A medical history of CVE was included as a composite marker to reflect overall cardiovascular risk burden. Pre-transplant hypertension was defined as documented hypertension before transplantation, based on ICD-10 codes. Diabetes was defined as documented diabetes, based on ICD-10 codes, or use of antidiabetic medication (metformin, sulfonylurea derivatives, SGLT2 inhibitors, DPP-4 inhibitors, GLP-1 receptor agonists, and insulin). Salt intake (g/day) was estimated from 24-h urinary sodium excretion [31]. Lifestyle parameters, including alcohol intake, and smoking status, were derived from self-reported questionnaires.

### Outcome measures

The primary outcome was the proportion of SOT recipients with suboptimal hypertension control. As there is no clear consensus about the optimal target values for SBP and DBP after SOT, a target value of ≤130/80 mmHg was applied. This decision was based on local, national and international guidelines, such as the Dutch national guidelines for cardiovascular risk management [32], the guidelines of the various SOT departments at the UMCG, the KDIGO guidelines [20], American guidelines for prevention, detection, and management of high blood pressure in adults [18], and two studies investigating different blood pressure target values [19, 21]. Hypertension was defined as a SBP >130 mmHg, a DBP >80 mmHg or current use of antihypertensive medication. Suboptimal hypertension control was defined as a SBP >130 mmHg or DBP >80 mmHg, regardless of antihypertensive treatment. The following medication were considered as antihypertensives: calcium channel antagonists, beta-blockers, angiotensin-converting enzyme-inhibitors (ACEi), angiotensin receptor blockers (ARB), alpha-blockers, loop diuretics, thiazide diuretics and potassium-sparing diuretics. The secondary outcome was the difference in suboptimal hypertension control between the transplantation programs (heart, liver, lung and kidney).

### Statistical analysis

Statistical analyses were performed using SPSS software (version 28.0, IBM Corporation, Armonk, NY) and R version 4.4.1. Normality of distribution was visually assessed using histograms and quartile-quartile plots. Continuous data were presented as means with standard deviations (SD) or medians with interquartile ranges (IQR), depending on data distribution, or in numbers with valid percentages for categorical data. Differences between groups were assessed using independent t-tests or one-way ANOVA for normally distributed variables, with Tukey’s HSD or, when variances were unequal, the Games–Howell *post hoc* test applied when applicable. For non-normally distributed variables, the Mann–Whitney U test or Kruskal–Wallis test was used, with Dunn’s *post hoc* test (Holm adjustment) applied when applicable. Categorical variables were compared using chi-square or Fisher’s exact tests with Bonferroni-corrected pairwise comparisons when applicable. To assess associations between recipient characteristics and blood pressure status, univariable and multivariable multinomial logistic regression analyses were performed. Blood pressure status was categorized as no hypertension (reference group), optimal hypertension control, and suboptimal hypertension control. Multivariable analyses were adjusted for age, sex, and transplantation type. To assess factors associated with antihypertensive treatment among hypertensive SOT recipients, additional univariable and multivariable binary logistic regression analyses were performed comparing treated versus untreated recipients with suboptimal control.

As optimal blood pressure thresholds after SOT are still debated in the literature, sensitivity analyses were performed using target values of ≤135/85 mmHg and ≤140/90 mmHg. Furthermore, because blood pressure measurements differed, and included extractions from EPD and protocolized measurements, a sensitivity analysis restricted to protocolized blood pressure measurements was performed to evaluate the robustness of our findings. To assess potential selection bias due to missing blood pressure data in the liver transplant population, baseline characteristics of included and excluded liver transplant recipients were compared. As CVE was analyzed as a composite outcome, the individual cardiovascular disease components (myocardial infarction, heart failure, peripheral vascular disease, and CVA/TIA) were analyzed separately to assess potential heterogeneity between conditions.

Finally, concordance between prescribed antihypertensive agents and 24-h urinary metabolite detection was assessed in a random sample of the population for selected antihypertensives. Metabolites of ACEi, ARB, and beta-blockers were evaluated through reversed-phase liquid chromatography coupled to high-resolution quadrupole-time-of-flight mass spectrometry in positive electrospray ionization and with SWATH data-independent acquisition. Recipients were classified as concordant, partially concordant, or non-concordant based on urinary metabolite detection relative to prescribed medication.

## Results

### Study population

Out of 1169 recipients with available data at 1-year post-transplantation included in the TransplantLines biobank and cohort study, 57 recipients (4.9%) were excluded because of missing data on SBP or DBP. This concerned one heart transplant recipient, 31 liver transplant recipients, 3 lung transplant recipients and 22 kidney transplant recipients. Consequently, 1112 SOT recipients were included in the current study, among which 37 heart, 119 liver, 162 lung and 794 kidney transplant recipients (Supplementary Figure S1). The mean age of this population was 57 ± 13 years, and 433 recipients (39%) were female. Mean SBP was 134 ± 16 mmHg and mean DBP was 78 ± 10 mmHg (Table 1; full version in Supplementary Table S2).

**TABLE 1:** Characteristics of the SOT recipient population

Characteristics	N	SOT recipients n = 1112
Demographics		
Age, years	1112	57 ± 13
Sex, n (%)	1112	​
Male	​	679 (61)
Female	​	433 (39)
BMI, kg/m2	1084	26.9 ± 4.4
Metabolic syndrome, n (%)	760	​
No	​	441 (58)
Yes	​	319 (42)
Transplantation type, n (%)	1112	​
Kidney	​	794 (71)
Liver	​	119 (11)
Lung	​	162 (15)
Heart	​	37 (3)
Blood pressure parameters		
Systolic blood pressure, mmHg	1112	134 ± 16
Diastolic blood pressure, mmHg	1112	78 ± 10
Pulse rate, freq/min	822	74 ± 13
Pulse pressure, mmHg	1112	55 ± 15
Mean arterial pressure, mmHg	1112	97 ± 10
Medical history		
Cardiovascular event, n (%)	1112	281 (25)
Diabetes, n (%)	1112	326 (29)
Pre-transplant hypertension	1112	40 (31)
Medication use		
Number of antihypertensives, n (%)	1112	​
0	​	261 (24)
1	​	347 (31)
>1	​	504 (45)

To assess potential selection bias in the liver transplant population, baseline characteristics of included and excluded liver transplant recipients were compared. Differences in baseline characteristics between groups were limited (Supplementary Table S3).

### Prevalence of hypertension and suboptimal hypertension control in SOT recipients

**FIGURE 1:**
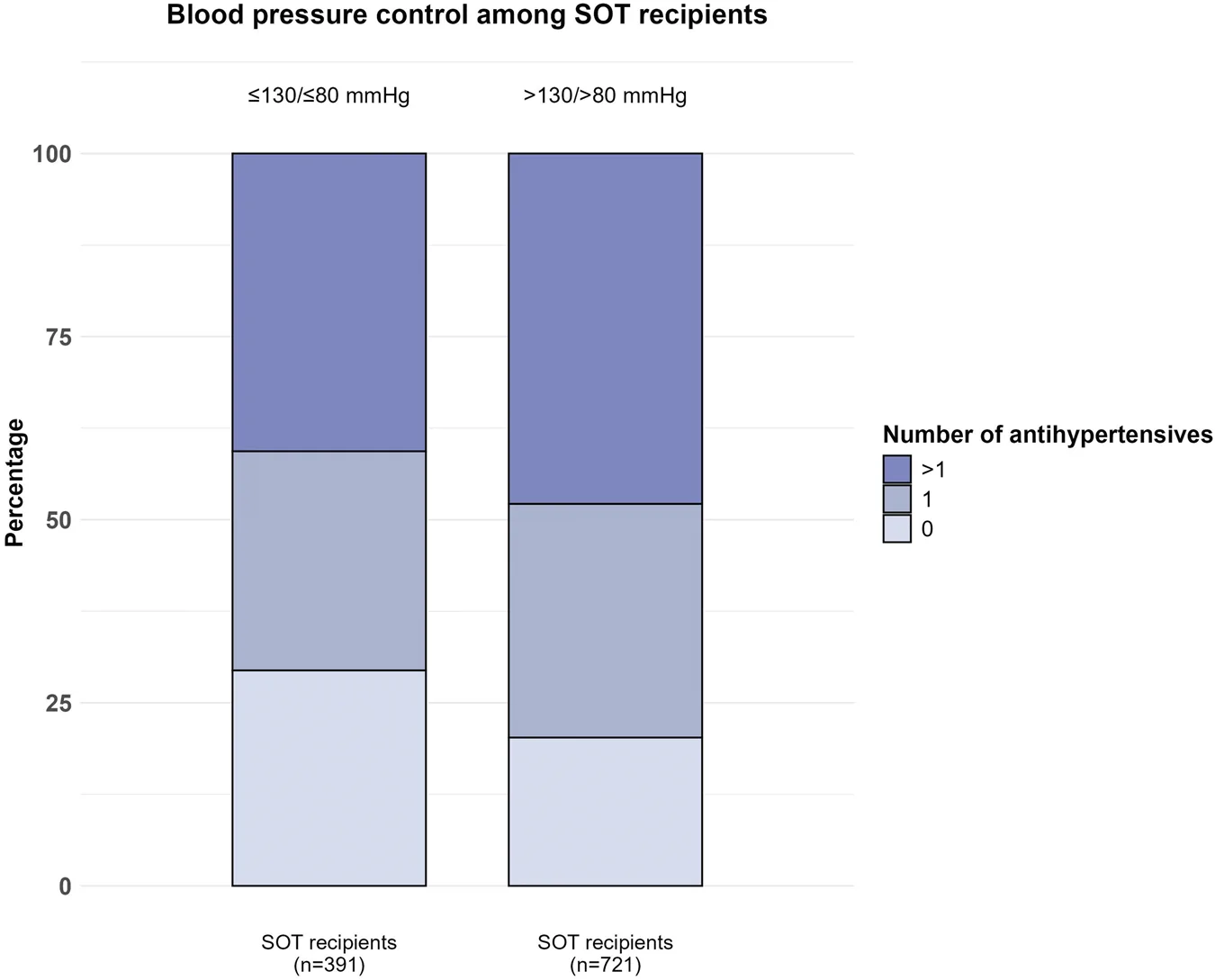
Distribution of number of antihypertensives across SOT recipients stratified according to the threshold of >130/>80 mmHg.

Of the SOT recipients, 997 (90%) had hypertension. Of the recipients with hypertension, 851 (85%) used antihypertensives. Within this hypertensive group, 721 (72%) had suboptimal hypertension control and 146 (20%) of them were untreated (Figure 1).

### Hypertension status and hypertension control in SOT recipients

SOT recipients with hypertension were more often male, had a higher BMI, had more frequently metabolic syndrome, had more often cardiovascular events in their medical history, had more often pre-transplant hypertension, had higher hematocrit and hemoglobin levels, and had a lower eGFR compared to recipients without hypertension. Recipients with suboptimal control had more frequently diabetes compared to recipients without hypertension. Additionally, recipients with suboptimal control were older, had a higher pulse pressure and had a higher mean arterial pressure (Supplementary Table S4).

In multivariable multinomial logistic regression, using recipients without hypertension as the reference group, several characteristics were independently associated with hypertension status (optimal or suboptimal).

Compared with recipients without hypertension, optimal hypertension control was independently associated with male sex (OR = 1.74; 95%CI1.10–2.76), higher BMI (OR = 1.11; 95%CI1.05–1.18), presence of metabolic syndrome (OR = 8.28; 95%CI3.84–17.84), larger waist circumference (OR = 1.04; 95%CI1.01–1.08), prior cardiovascular events (OR = 2.38; 95%CI1.15–4.92), pre-transplant hypertension (OR = 2.82; 95%CI1.44-5.51) and lower HDL-cholesterol levels (OR = 0.52; 95%CI0.30–0.91) (Supplementary Table S5).

Compared with recipients without hypertension, suboptimal hypertension control was independently associated with older age (OR = 1.03; 95%CI1.01–1.04), male sex (OR = 1.98; 95%CI1.31–3.01), higher BMI (OR = 1.13; 95%CI1.07–1.19), presence of metabolic syndrome (OR = 9.05; 95%CI4.38–18.69), larger waist circumference (OR = 1.04; 95%CI1.01–1.07), prior cardiovascular events (OR = 2.08; 95%CI1.04–4.14), presence of diabetes (OR = 1.99; 95%CI1.18–3.36), pre-transplant hypertension (OR = 2.02; 95%CI1.07-3.84), higher levels of total cholesterol (OR = 1.23; 95%CI1.01–1.51), higher levels of triglycerides (OR = 1.33; 95%CI1.04–1.69), higher levels of creatinine (OR = 2.07; 95%CI1.05–4.08), and a lower eGFR (OR = 0.99; 95%CI0.97–1.00) (Supplementary Table S5).

### Treatment status of suboptimally controlled hypertension in SOT recipients

Suboptimal control was observed in 721 (72%) of recipients with hypertension, of whom 146 (20%) were not receiving antihypertensive treatment. These participants were younger, more often female, and had a lower BMI. They also had a higher pulse rate, lower pulse pressure, higher mean arterial pressure, lower salt intake, a lower prevalence of cardiovascular events, and a lower prevalence of pre-transplant hypertension compared with those who received treatment. Additionally, they used less often prednis(ol)on. In terms of metabolic and renal parameters, they showed lower hematocrit levels, lower hemoglobin levels, higher LDL-cholesterol values and higher eGFR values (Supplementary Table S6).

In multivariable logistic regression analyses several factors were independently associated with treatment: a higher age (OR = 1.03; 95%CI1.01–1.05), male sex (OR = 1.55; 95%CI1.03–2.34), higher salt intake (OR = 1.07; 95%CI1.00–1.14), medical history of cardiovascular events (OR = 2.06; 95%CI1.14–3.95), higher levels of glucose (OR = 1.16; 95%CI1.04–1.31), and lower eGFR values (OR = 0.99; 95%CI0.97–1.00) (Supplementary Table S7).

### Differences between the solid organ programs

**FIGURE 2:**
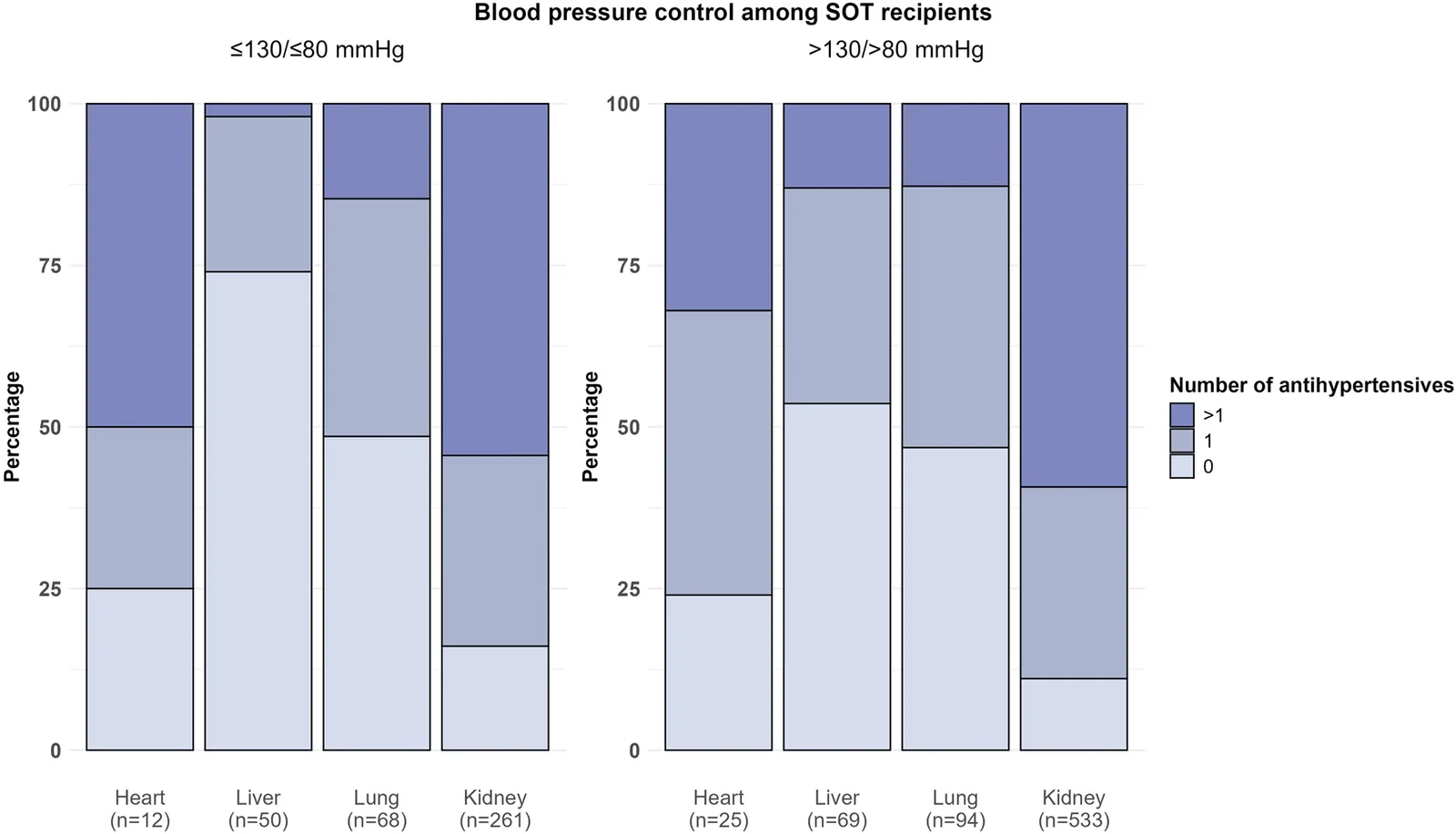
Distribution of number of antihypertensives across heart, liver, lung and kidney transplant recipients stratified according to the threshold of >130/>80 mmHg.

Comparing the different SOT transplantation programs, hypertension prevalence was different between the groups (p < 0.001), with hypertension present in 92% of heart, 69% of liver, 80% of lung and 95% of kidney transplant recipients. Suboptimal hypertension control was observed in 74% heart, 84% liver, 73% lung and 71% kidney transplant recipients, with no significant difference between groups (p = 0.09). However, the proportion of untreated recipients within the suboptimal hypertension control group varied significantly between organ groups (p < 0.001): 24% of heart, 54% of liver, 47% of lung, and 11% of kidney transplant recipients, with suboptimal hypertension control, remained untreated. Additionally, 32% of heart, 13% of liver, 12% of lung and 59% of kidney transplant recipients with suboptimal treatment, received multiple antihypertensive agents (Figure 2).

Kidney transplant recipients were more likely to have both optimally and suboptimally controlled hypertension compared with liver and lung transplant recipients (Supplementary Table S5). No significant differences were observed between kidney and heart transplant recipients.

### Treatment status of suboptimally controlled hypertension across organ programs

Kidney transplant recipients without treatment were primarily younger, more frequently women, had less often diabetes and had fewer cardiovascular events in their medical history (Supplementary Table S8). Liver transplant recipients without treatment were characterized by a lower frequency of metabolic syndrome, lower frequency of pre-transplant hypertension and lower salt intake (Supplementary Table S9). Untreated lung transplant recipients were younger compared to those treated (Supplementary Table S10). No significant differences were found in the heart transplant subgroup (Supplementary Table S11).

Kidney transplant recipients were more likely to receive antihypertensive treatment compared to liver and lung transplant recipients (Supplementary Table S7). Heart transplant recipients did not differ significantly from kidney transplant recipients.

### Sensitivity analyses

Different blood pressure cut-off values (≤135/85 mmHg and ≤140/90 mmHg) were applied in sensitivity analyses. The proportion of recipients reaching these target values increased compared to the primary threshold of ≤130/80 mmHg. Using a target cut-off of ≤135/85 mmHg, hypertension occurred in 957 (86%) of SOT recipients and suboptimal control was present in 524 (55%) SOT recipients with hypertension (Supplementary Figure S2). Across transplant groups, the proportion of SOT recipients with suboptimal hypertension control ranged from 52% to 76% (Supplementary Figure S3).

**FIGURE 3:**
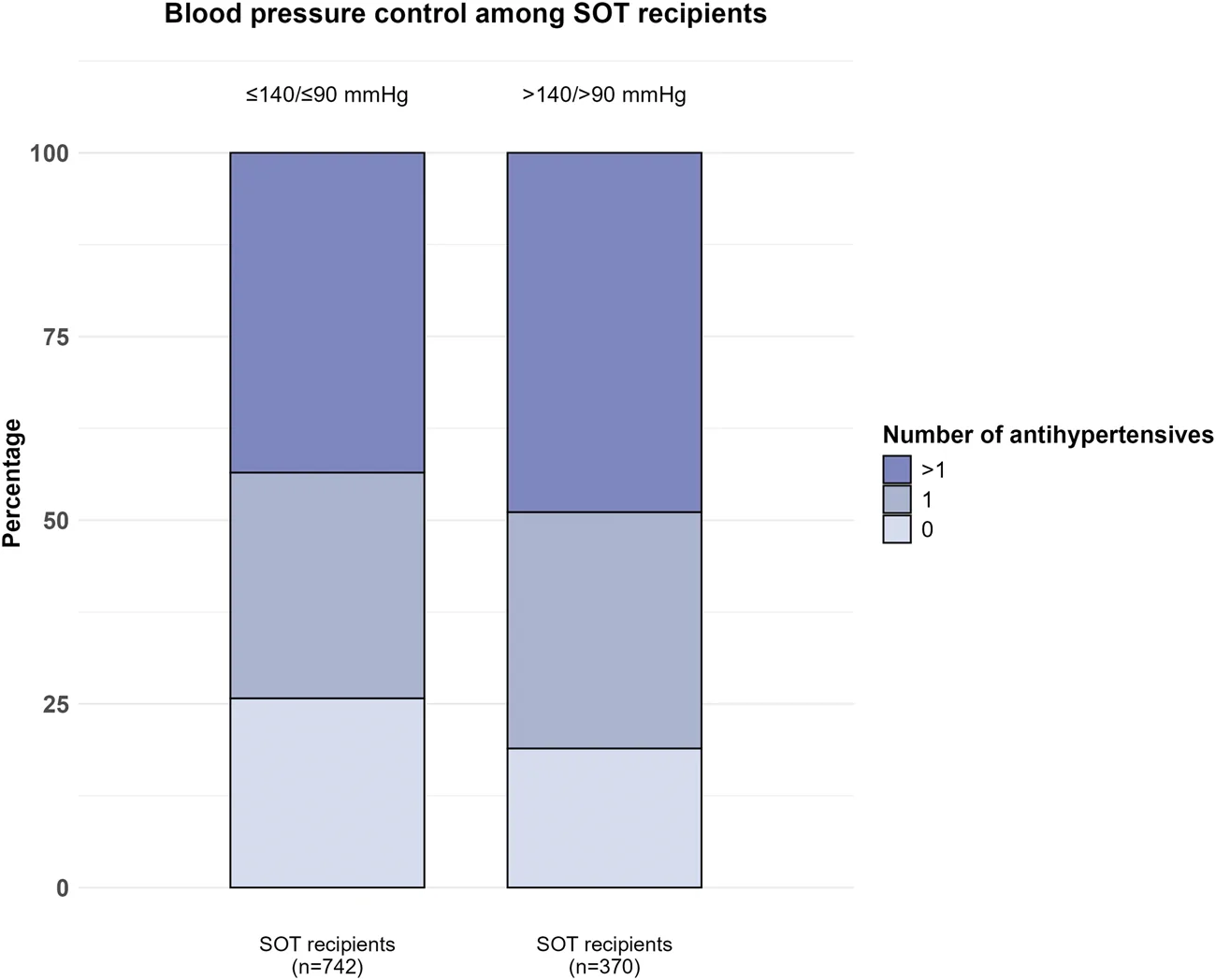
Distribution of number of antihypertensives across SOT recipients stratified according to the threshold of >140/>90 mmHg.

Using a target cut-off of ≤140/90 mmHg, hypertension occurred in 921 (83%) of SOT recipients and suboptimal control was present in 370 (40%) SOT recipients with hypertension (Figure 3). Across transplant groups, the proportion of SOT recipients with suboptimal hypertension control ranged from 44% to 62% (Supplementary Figure S4).

To address potential heterogeneity in blood pressure measurements, an additional sensitivity analysis was performed restricted to recipients with protocolized study visit-based blood pressure measurements (Supplementary Table S12). In the primary analysis, suboptimal hypertension control was present in 721 of 997 hypertensive SOT recipients (72%). Findings remained consistent when restricting the analysis to recipients assessed with protocolized study visit-based measurements (298/386, 77%).

Because CVE was analyzed as a composite outcome, separate sensitivity analyses were performed for the individual cardiovascular disease components (myocardial infarction, heart failure, peripheral vascular disease, and CVA/TIA) to assess potential heterogeneity. These analyses showed broadly comparable directions of association to those observed for the composite CVE outcome. However, confidence intervals were wider and statistical significance was less consistent across individual cardiovascular conditions (Supplementary Tables S13, 14).

### Antihypertensive medication adherence

In a randomly selected subgroup of 156 recipients, metabolites of selected antihypertensive agents (ACEi, ARB, and beta-blockers) were analyzed in 24-h urine samples. Of these, 60 recipients used no antihypertensive medication according to medication lists, consistent with urinary metabolite measurements. Of the 96 recipients prescribed one or more ACEi, ARB, or beta-blockers, 88 (92%) showed concordance between prescribed medication and urinary metabolite detection, whereas 4 (4%) showed partial concordance and 4 (4%) showed no concordance (Supplementary Table S15).

## Discussion

In this large cross-sectional study among SOT recipients, hypertension was highly prevalent. Among SOT recipients, 997 (90%) had hypertension, and of them 721 (72%) had suboptimal hypertension control. Importantly, findings remained consistent in a sensitivity analysis restricted to protocolized blood pressure measurements. In addition, the observed burden of suboptimal hypertension control depended on the blood pressure threshold applied. While 72% of hypertensive recipients had suboptimal control using the primary threshold of ≤130/80 mmHg, suboptimal control remained common even when applying less stringent thresholds of ≤135/85 mmHg and ≤140/90 mmHg (55% and 40%, respectively) in sensitivity analyses. These findings suggest that suboptimal control of hypertension in SOT recipients remains clinically relevant irrespective of the threshold used. Similar prevalence rates have been reported in another transplant population, however the proportion of suboptimally control hypertension in our cohort was higher [13, 33]. Other studies in transplant populations about hypertension control remain limited. In a population with chronic kidney disease a high prevalence of uncontrolled hypertension has also been described [34]. Maintaining adequate blood pressure control in the SOT population is of significant importance, given the high burden of multimorbidity and elevated cardiovascular risk.

In this study was seen that recipients without hypertension had a lower risk profile (younger age, better profile of cardiovascular parameters, higher eGFR, less frequently pre-transplant hypertension and less frequently metabolic syndrome, cardiovascular events or diabetes in their medical history), as expected. Recipients with suboptimal control but a comparable low-risk profile were more often left untreated, likely because their profile was similar to that of recipients without hypertension. Recipients with a more typical high-risk profile (more frequent metabolic syndrome, and a history of cardiovascular disease) were more likely to be treated in the suboptimally controlled group, reflecting practices seen in the general population. Mildly elevated blood pressure in healthy individuals is often managed initially with lifestyle interventions, reserving pharmacological treatment for those at higher cardiovascular risk [32]. However, given the elevated cardiovascular risk among transplant recipients, pharmacological treatment is crucial even in seemingly low-risk patients. Recipients who remain untreated, or who are on monotherapy despite suboptimal control, should be considered for treatment intensification. Age and prior cardiovascular events were associated with both suboptimal hypertension control and with receiving antihypertensive agents. This was expected, as hypertension becomes more prevalent and resistant with older age and cardiovascular history [35]. These risk factors are likely closely monitored by physicians, explaining their association with treatment in the suboptimally controlled group. Immunosuppressive therapy, particularly calcineurin inhibitors and corticosteroids, has been recognized as a contributor to post-transplant hypertension [36]. However, no independent association between immunosuppressive therapy and hypertension status or antihypertensive treatment was observed in our analyses. This may partly reflect limited contrast in exposure within our cohort, as all recipients were assessed 1 year after transplantation, used immunosuppressive therapy, and hypertension was highly prevalent.

Furthermore, salt intake was high in this cohort, suggesting that, in addition to pharmacological treatment given the high cardiovascular risk in transplant recipients, complementary lifestyle interventions may help optimize blood pressure control.

Stratification by transplant type showed that hypertension was most prevalent among heart and kidney recipients. Suboptimal control was similar across groups, but treatment patterns differed. Most kidney and heart recipients received antihypertensive therapy, often with multiple agents, while lung and liver recipients were less frequently treated, with many either untreated or managed with monotherapy despite being above target. Heart and kidney are primary target organs of hypertension and have a more direct role in blood pressure regulation [37], making strict treatment crucial for graft preservation. This can explain the higher treatment rates in heart and kidney recipients in comparison to liver and lung recipients. Additionally, mean survival differs between organ recipient groups making cardiovascular control particularly important for those with longer expected survival, as cardiovascular complications become more relevant over the long term. Notably, mean survival is lowest among lung transplant recipients [38]. Nevertheless, effective blood pressure control remains essential to reduce long-term morbidity and mortality. Compared to lung and liver recipients, many kidney and heart recipients remained hypertensive despite multi-drug therapy. This reflects the complexity of hypertension control in SOT recipients, influenced by (interactions with) immunosuppressive drugs, renal vascular disease, and possibly resistant hypertension [39, 40]. Non-adherence could also contribute, but we lacked comprehensive adherence data. In heart transplant recipients specifically, frequent follow-up and invasive procedures (e.g., routine biopsies according to local protocols) in the first year may delay blood pressure optimization [41, 42]. Better control may be achieved later, although definitive conclusions could not be drawn given the exploratory nature of the analyses in this relatively small heart-transplant subgroup. Nevertheless, heart transplant recipients were included because this study evaluated different solid organ transplant populations, of which heart transplantation represents one subgroup.

Strengths of this study include its large sample size and the inclusion of four distinct SOT groups, enabling broad comparisons across transplant populations. Limitations include the single-center and cross-sectional design, which precludes causal inference, and the inability to assess blood pressure trends over time. Medication changes due to side effects or lack of efficacy were not recorded. Another limitation of this study is the absence of systematic medication adherence data. Non-adherence is a recognized contributor to uncontrolled hypertension and may partly explain persistent elevated blood pressure [43], particularly among recipients receiving multiple antihypertensive agents. However, in a randomly selected subgroup of recipients, concordance between prescribed medication and urinary metabolite detection was high (>90%). Nevertheless, these measurements were analyzed in a subset of recipients and did not include all antihypertensive drug classes. Therefore, non-adherence cannot be excluded as a contributor to suboptimal blood pressure control. Additionally, phenomena such as masked hypertension and white coat hypertension may have led to under- or overestimation of blood pressure control rates [44]. Specifically, masked hypertension may have resulted in underestimation of the prevalence of hypertension and suboptimal blood pressure control, whereas white coat hypertension may have contributed to overestimation. This limitation is particularly relevant because most blood pressure measurements in the present study were based on office blood pressure assessments rather than 24-h ambulatory blood pressure monitoring (ABPM), which can also account for nocturnal hypertension. In kidney transplant recipients, approximately 32% have been reported to experience masked hypertension and 6% white coat hypertension, highlighting the potential for misclassification when relying solely on office blood pressure measurements [44]. Furthermore, blood pressure measurements were unavailable for a disproportionate number of liver transplant recipients. Although baseline characteristics between included and excluded liver transplant recipients were largely comparable, excluded recipients had a higher prevalence of prior cardiovascular events, and the possibility of selection bias or non-random missingness cannot be excluded. A minor limitation is that, among participants classified as using a single antihypertensive medication, some may have been prescribed the drug for reasons other than hypertension. While this affects only a small proportion of the cohort, it introduces some uncertainty in the classification of antihypertensive use for these individuals. This could also be the case for the definition of diabetes, as this was defined based on ICD-10 codes or the use of antidiabetic medication. A limited number of antidiabetic medication may also be prescribed for indications other than diabetes. Finally, as the heart-transplant program represents a relative small subgroup, analyses in heart transplant recipients should be considered exploratory.

In conclusion, hypertension and suboptimal hypertension control remain highly prevalent among SOT recipients. Although most SOT recipients with suboptimal control were treated, target blood pressure was often not reached, even with multi-drug regimens. Given the elevated cardiovascular risk in this population, these findings highlight the importance of careful blood pressure monitoring and individualized evaluation of hypertension control. It is essential that recipients with suboptimal hypertension control receive appropriate therapy and that treatment is intensified for those suboptimally controlled. Additionally, lifestyle interventions such as dietary sodium restrictions could be considered. Finally, recipients on multiple antihypertensive agents may benefit from further assessment of factors potentially contributing to suboptimal blood pressure control, including therapy-resistant hypertension or non-adherence. Improving hypertension control in SOT recipients may represent an important opportunity to reduce cardiovascular risk and mortality.

## Data Availability

The data analyzed in this study is subject to the following licenses/restrictions: Due to patient confidentiality and privacy restrictions, individual participant data from the TransplantLines Biobank and Cohort Study cannot be made publicly available, as public data sharing was not included in the informed consent forms. However, the data may be made available to qualified researchers upon reasonable request and approval by the TransplantLines Scientific Committee. Requests to access these datasets should be directed to datarequest.transplantlines@umcg.nl.
