# Correction: Blood pressure control after solid organ transplantation: opportunities for optimizing care

**DOI:** 10.3389/ti.2026.17659

**Published:** 2026-09-01

**Authors:** Transplant International Production Office

**Affiliations:** Frontiers Media SA, Lausanne, Switzerland

**Keywords:** blood pressure management, hypertension, solid organ transplantation, suboptimal treatment, uncontrolled hypertension

**The TransplantLines investigators** was omitted in the author list of the published article. The correct author list reads:

A. M. Posthumus, T. J. Knobbe, D. Kremer, M. F. Eisenga, J. S. F. Sanders, S. P. Berger, C. Smit, F. Klont, C. T. Gan, E. A. M. Verschuuren, K. Damman, M. H. de Borst, H. Blokzijl, S. J. L. Bakker and V. E. de Meijer on behalf of The TransplantLines investigators.

The original version of this article has been updated.

