# Supplementary material for: Blood pressure control after solid organ transplantation: opportunities for optimizing care
Source: Transpl Int. 2026 Jul 30;39:16565. doi: 10.3389/ti.2026.16565 (PMC13468530; doi:10.3389/ti.2026.16565)
Supplement: Supplementary file 1 [file DataSheet1.docx]

**SUPPLEMENTARY MATERIAL**

Table S1: STrengthening the Reporting of OBservational studies in Epidemiology (STROBE) statement

|  | Item No. | Recommendation | Page  No. |
| --- | --- | --- | --- |
| **Title and abstract** | 1 | (*a*) Indicate the study’s design with a commonly used term in the title or the abstract | See Abstract |
|  |  | (*b*) Provide in the abstract an informative and balanced summary of what was done and what was found | See Abstract |
| Introduction | | | |
| Background/rationale | 2 | Explain the scientific background and rationale for the investigation being reported | See Introduction par 1-4 |
| Objectives | 3 | State specific objectives, including any prespecified hypotheses | See Introduction par 4 |
| Methods | | | |
| Study design | 4 | Present key elements of study design early in the paper | See Methods par 1 |
| Setting | 5 | Describe the setting, locations, and relevant dates, including periods of recruitment, exposure, follow-up, and data collection | See Methods par 1 |
| Participants | 6 | (*a*) Give the eligibility criteria, and the sources and methods of selection of participants | See Methods par 1 and Figure S1 |
|  |  | (*b*) *Cohort study*—For matched studies, give matching criteria and number of exposed and unexposed  *Case-control study*—For matched studies, give matching criteria and the number of controls per case | NA |
| Variables | 7 | Clearly define all outcomes, exposures, predictors, potential confounders, and effect modifiers. Give diagnostic criteria, if applicable | See Methods par 2 and 3 |
| Data sources/ measurement | 8* | For each variable of interest, give sources of data and details of methods of assessment (measurement). Describe comparability of assessment methods if there is more than one group | See Methods par 2 |
| Bias | 9 | Describe any efforts to address potential sources of bias | See Methods par 4 |
| Study size | 10 | Explain how the study size was arrived at | See Results par 1 and Figure S1 |
| Quantitative variables | 11 | Explain how quantitative variables were handled in the analyses. If applicable, describe which groupings were chosen and why | See Methods par 2 |
| Statistical methods | 12 | (*a*) Describe all statistical methods, including those used to control for confounding | See Methods par 4 and Results |
|  |  | (*b*) Describe any methods used to examine subgroups and interactions | See Methods par 4 |
|  |  | (*c*) Explain how missing data were addressed | See footnotes Table S2 |
|  |  | (*d*) *Cross-sectional study*—If applicable, describe analytical methods taking account of sampling strategy | See Methods par 4 |
|  |  | (*e*) Describe any sensitivity analyses | See Methods par 4 and Results par 7 |
| **Results** |  |  |  |
| Participants | 13* | (a) Report numbers of individuals at each stage of study—eg numbers potentially eligible, examined for eligibility, confirmed eligible, included in the study, completing follow-up, and analysed | See Figure S1 |
|  |  | (b) Give reasons for non-participation at each stage | See Figure S1 |
|  |  | (c) Consider use of a flow diagram | See Figure S1 |
| Descriptive data | 14* | (a) Give characteristics of study participants (eg demographic, clinical, social) and information on exposures and potential confounders | See Results par 1,  Table 1 and Table S2 |
|  |  | (b) Indicate number of participants with missing data for each variable of interest | See Table 1 and Table S2 |
|  |  | (c) *Cohort study*—Summarise follow-up time (eg, average and total amount) | NA |
| Outcome data | 15* | *Cross-sectional study—*Report numbers of outcome events or summary measures | See Results par 2-6, Tables and Figures |
| Main results | 16 | (*a*) Give unadjusted estimates and, if applicable, confounder-adjusted estimates and their precision (eg, 95% confidence interval). Make clear which confounders were adjusted for and why they were included | See Results, Methods par 4, Tables and Figures |
|  |  | (*b*) Report category boundaries when continuous variables were categorized | See Methods par 3 and 4 |
|  |  | (*c*) If relevant, consider translating estimates of relative risk into absolute risk for a meaningful time period | NA |
| Other analyses | 17 | Report other analyses done—eg analyses of subgroups and interactions, and sensitivity analyses | See Methods par 4 and Results par 7 |
| **Discussion** |  |  |  |
| Key results | 18 | Summarise key results with reference to study objectives | Discussion par 6 |
| Limitations | 19 | Discuss limitations of the study, taking into account sources of potential bias or imprecision. Discuss both direction and magnitude of any potential bias | Discussion par 5 |
| Interpretation | 20 | Give a cautious overall interpretation of results considering objectives, limitations, multiplicity of analyses, results from similar studies, and other relevant evidence | Discussion par 1-6 |
| Generalisability | 21 | Discuss the generalisability (external validity) of the study results | See Discussion par 1 |
| Other information |  |  |  |
| Funding | 22 | Give the source of funding and the role of the funders for the present study and, if applicable, for the original study on which the present article is based | See Disclosures |

Abbreviation: par; paragraph.

Figure S1: flowchart of SOT recipient inclusion from the TransplantLines Biobank and Cohort study

Abbreviations: SOTR; solid organ transplant recipients, SBP; systolic blood pressure, DBP; diastolic blood pressure.

Table S2: characteristics of the SOT recipient population

|  | N | SOT recipients  n = 1112 |
| --- | --- | --- |
| **Demographics** |  |  |
| Age, years | 1112 | 57±13 |
| Sex, n(%) | 1112 |  |
| Male |  | 679 (61) |
| Female |  | 433 (39) |
| BMI, kg/m2 | 1084 | 26.9±4.4 |
| Metabolic syndrome, n(%) | 760 |  |
| No |  | 441 (58) |
| Yes |  | 319 (42) |
| Waist circumference, cm | 446 |  |
| Male |  | 100±12 |
| Female |  | 93±12 |
| Transplantation type, n(%) | 1112 |  |
| Kidney |  | 794 (71) |
| Liver |  | 119 (11) |
| Lung |  | 162 (15) |
| Heart |  | 37 (3) |
| **Blood pressure parameters** |  |  |
| Systolic blood pressure, mmHg | 1112 | 134±16 |
| Diastolic blood pressure, mmHg | 1112 | 78±10 |
| Pulse rate, freq/min | 822 | 74±13 |
| Pulse pressure, mmHg | 1112 | 55±15 |
| Mean arterial pressure, mmHg | 1112 | 97±10 |
| **Lifestyle parameters** |  |  |
| Salt intake, g/day | 925 | 8.5 [6.3-11.1] |
| Alcohol intake a week, n(%) | 471 |  |
| <7 units |  | 401 (85) |
| ≥7 units |  | 70 (15) |
| Smoking, n(%) | 872 |  |
| Non-smoker |  | 544 (62) |
| Ex-smoker |  | 252 (29) |
| Current |  | 76 (9) |
| **Medical history** |  |  |
| Cardiovascular event, n(%) | 1112 | 281 (25) |
| Diabetes, n(%) | 1112 | 326 (29) |
| Pre-transplant hypertension | 1112 | 40 (31) |
| **Medication use** |  |  |
| Number of antihypertensives, n(%) | 1112 |  |
| 0 |  | 261 (24) |
| 1 |  | 347 (31) |
| 2 |  | 265 (24) |
| 3 |  | 158 (14) |
| 4 |  | 72 (6) |
| 5 |  | 7 (<1) |
| 6 |  | 2 (<1) |
| Group of antihypertensives, n(%) |  |  |
| Calcium channel antagonists | 1112 | 471 (42) |
| ARB | 1112 | 90 (8) |
| ACE-inhibitor | 1112 | 145 (13) |
| Beta-blockers | 1112 | 572 (51) |
| Alpha-blocker | 1112 | 164 (15) |
| Loop diuretics | 1112 | 152 (14) |
| Thiazide diuretics | 1112 | 54 (5) |
| Potassium-sparing diuretics | 1112 | 25 (2) |
| Immunosuppressives |  |  |
| Tacrolimus | 1112 | 1040 (94) |
| Ciclosporin | 1112 | 52 (5) |
| Mycophenolic acid | 1112 | 10 (1) |
| Mycophenolate mofetil | 1112 | 873 (79) |
| Azathioprine | 1112 | 49 (4) |
| Everolimus | 1112 | 96 (9) |
| Sirolimus | 1112 | 14 (1) |
| Predniso(lo)ne | 1112 | 982 (88) |
| **Laboratory parameters** |  |  |
| **Blood values** |  |  |
| Hematocrit, L/L | 1101 | 0.40±0.05 |
| Hemoglobin, mmol/L | 1102 | 8.0±1.1 |
| Total cholesterol, mmol/L | 1049 | 4.6±1 |
| LDL cholesterol, mmol/L | 1045 | 2.6 [2.0-3.2] |
| HDL cholesterol, mmol/L | 1046 | 1.3 [1.1-1.6] |
| TC/HDL cholesterol ratio | 1046 | 3.2 [2.6-4.1] |
| Triglycerides, mmol/L | 1046 | 1.71 [1.26-2.32] |
| HbA1c, mmol/mol | 1054 | 41 [36-46] |
| Glucose, mmol/L | 1098 | 5.9 [5.3-7.0] |
| CRP, mg/L | 1096 | 2.0 [0.8-5.0] |
| eGFR, ml/min/1.73m^2^ | 1109 | 54±19 |
| Creatinine, µmol/L | 1110 | 120 [97-147] |
| **24 hour urine values** |  |  |
| Sodium, mmol/L | 925 | 62 [45-84] |
| Estimated urine output over | 928 | 2337±869 |
| 24 hours, mL |  |  |

Abbreviations: ARB; angiotensin receptor blockers, ACE; angiotensin-converting enzyme, LDL; low-density lipoprotein, HDL; high density lipoprotein, TC; total cholesterol, CRP; C-reactive protein, eGFR; estimated glomerular filtration rate.

N represents the sample size for each variable. Pairwise exclusion was used to handle missing data.

Table S3: Baseline characteristics of included and excluded liver transplant recipients

|  | Excluded  n= 31 | Included  n= 119 | P-value |
| --- | --- | --- | --- |
| **Demographics** |  |  |  |
| Age, years | 59±11 | 59±11 | 0.07 |
| Sex, n(%) |  |  | 0.6 |
| Male | 19 (61) | 65 (55) |  |
| Female | 12 (39) | 54 (45) |  |
| BMI, kg/m2 | 27.3±4.6 | 27.3±4.6 | 0.1 |
| Metabolic syndrome, n(%) |  |  | <1.0 |
| No | 13 (65) | 57 (67) |  |
| Yes | 7 (35) | 28 (33) |  |
| **Life style parameters** |  |  |  |
| Salt intake, g/day | 8.2 [5.3, 11.3] | 9.2 [6.6, 11.7] | 0.4 |
| Alcohol intake a week, n(%) |  |  | 0.2 |
| <7 units | 4 (80) | 48 (100) |  |
| ≥7 units | 1 (20) | 0 (0) |  |
| Smoking, n(%) |  |  | 0.8 |
| Non-smoker | 18 (67) | 58 (60) |  |
| Ex-smoker | 7 (26) | 29 (30) |  |
| Smoker | 2 (7) | 9 (10) |  |
| **Medical history** |  |  |  |
| Cardiovascular event, n(%) | 8 (26) | 11 (9) | 0.03 |
| Diabetes, n(%) | 14 (45) | 39 (33) | 0.3 |
| Pre-transplant hypertension, n(%) | 5 (16) | 16 (13) | 0.9 |
| **Medication use** |  |  |  |
| Number of antihypertensives, n(%) |  |  | 0.4 |
| 0 | 18 (58) | 74 (62) |  |
| 1 | 8 (26) | 35 (29) |  |
| >1 | 5 (16) | 10 (9) |  |
| Immunosuppressives |  |  |  |
| Tacrolimus | 29 (94) | 112 (94) | <1.0 |
| Ciclosporin | 0 (0) | 0 (0) | NA |
| mTOR inhibitors | 12 (39) | 42 (35) | 0.9 |
| Antiproliferation agents | 16 (52) | 60 (50) | <1.0 |
| Prednis(ol)on | 7 (23) | 18 (15) | 0.5 |
| **Laboratory parameters** |  |  |  |
| Hematocrit, L/L | 0.38±0.06 | 0.38±0.06 | 0.9 |
| Hemoglobin, mmol/L | 7.72±1.35 | 7.69±1.23 | 0.9 |
| LDL cholesterol, mmol/L | 2.5 [2.0-3.1] | 2.7 [2.1-3.2] | 0.6 |
| HDL cholesterol, mmol/L | 1.3 [0.9-1.5] | 1.2 [1.0-1.5] | 0.4 |
| TC/HDL cholesterol ratio | 3.8 [3.1-4.8] | 3.4 [2.7-4.6] | 0.4 |
| Triglycerides, mmol/L | 1.82 [1.40-2.17] | 1.52 [0.98-2.15] | 0.2 |
| Total cholesterol, mmol/L | 6.1±8.7 | 4.6±1.2 | 0.07 |
| HbA1c, mmol/mol | 39 [34, 42] | 37 [33-42] | <1.0 |
| Glucose, mmol/L | 6.0 [5.5-7.5] | 5.9 [5.3-7.3] | 0.5 |
| CRP, mg/L | 2.1 [0.8-4.9] | 2.5 [1.1-6.3] | 0.7 |
| eGFR, ml/min/1.73m^2^ | 67±26 | 67±24 | <1.0 |

Abbreviations: LDL; low-density lipoprotein, HDL; high density lipoprotein, TC; total cholesterol, CRP; C-reactive protein, eGFR; estimated glomerular filtration rate.

Table S4: Characteristics of SOT recipients, stratified according to hypertension (control) status.

|  | No hypertension  n= 115 | Optimal  control  n= 276 | Suboptimal  control  n= 721 | Overall p-value |
| --- | --- | --- | --- | --- |
| **Demographics** |  |  |  |  |
| Age, years | 53±14^a^ | 55±15^b^ | 58±13^ab^ | <0.001 |
| Sex, n(%) |  |  |  | <0.001 |
| Male | 51 (44)^ab^ | 168 (61)^a^ | 460 (64)^b^ |  |
| Female | 64 (56)^ab^ | 108 (39)^a^ | 261 (36)^b^ |  |
| BMI, kg/m2 | 24.6±4.0^ab^ | 27.0±4.7^a^ | 27.2±4.2^b^ | <0.001 |
| Metabolic syndrome, n(%) |  |  |  | <0.001 |
| No | 93 (91)^ab^ | 92 (54)^a^ | 256 (52)^b^ |  |
| Yes | 9 (9)^ab^ | 78 (46)^a^ | 232 (48)^b^ |  |
| Waist circumference, cm |  |  |  |  |
| Male | 93±11^ab^ | 100±12^a^ | 101±12^b^ | 0.03 |
| Female | 87±12 | 94±14 | 94±12 | 0.05 |
| Transplantation type, n(%) |  |  |  |  |
| Kidney | 42 (5)^ab^ | 219 (28)^ac^ | 533 (67)^bc^ | <0.001 |
| Liver | 37 (31)^ab^ | 13 (11)^ac^ | 69 (58)^bc^ | <0.001 |
| Lung | 33 (20)^a^ | 35 (22)^b^ | 94 (58)^ab^ | <0.001 |
| Heart | 3 (8)^ab^ | 9 (24)^ac^ | 25 (68)^bc^ | <0.001 |
| **Blood pressure parameters** |  |  |  |  |
| Systolic blood pressure, mmHg | 118±9^ab^ | 121±8^ac^ | 141±14^bc^ | <0.001 |
| Diastolic blood pressure, mmHg | 72±7^a^ | 71±7^b^ | 82±10^ab^ | <0.001 |
| Pulse rate, freq/min | 77±11^a^ | 74±12 | 73±13^a^ | 0.02 |
| Pulse pressure, mmHg | 46±9^ab^ | 49±9^ac^ | 60±16^bc^ | <0.001 |
| Mean arterial pressure, mmHg | 87±7^a^ | 88±6^b^ | 102±8^ab^ | <0.001 |
| **Life style parameters** |  |  |  |  |
| Salt intake, g/day | 9.0 [6.6-10.4] | 7.9 [6.2-10.3] | 8.7 [6.3-11.6] | 0.07 |
| Alcohol, units a week, n(%) |  |  |  | 0.01 |
| <7 units | 48 (94) | 91 (91) | 262 (82) |  |
| ≥7 units | 3 (6) | 9 (9) | 58 (18) |  |
| Smoking, n(%) |  |  |  | 0.5 |
| Non-smoker | 62 (70) | 139 (61) | 343 (62) |  |
| Ex-smoker | 22 (25) | 65 (30) | 165 (30) |  |
| Current | 5 (6) | 24 (11) | 47 (9) |  |
| **Medical history** |  |  |  |  |
| Cardiovascular event, n(%) | 13 (11)^ab^ | 76 (28)^a^ | 192 (27)^b^ | 0.001 |
| Diabetes, n(%) | 21 (18)^a^ | 73 (26) | 232 (32)^a^ | 0.005 |
| Pre-transplant hypertension, n(%) | 13 (11)^ab^ | 104 (38)^a^ | 223 (31)^b^ | <0.001 |
| **Medication** |  |  |  |  |
| Medication use, n(%) |  |  |  |  |
| Immunosuppressives |  |  |  |  |
| Tacrolimus | 108 (94) | 258 (94) | 674 (94) | <1.0 |
| Ciclosporin | 4 (4) | 11 (4) | 36 (5) | 0.7 |
| mTOR inhibitors | 14 (12) | 17 (6) | 79 (11) | 0.05 |
| Antiproliferation agents | 91 (79) | 242 (88) | 596 (83) | 0.07 |
| Prednis(ol)on | 79 (69)^ab^ | 259 (94)^a^ | 644 (89)^b^ | <0.001 |
| **Medication use** |  |  |  |  |
| Number of antihypertensives, n(%) |  |  |  | <0.001 |
| 0 | 115 (100)^ab^ | 0 (0)^ac^ | 146 (20)^bc^ |  |
| 1 | 0 (0)^ab^ | 117 (42)^ac^ | 230 (32)^bc^ |  |
| >1 | 0 (0)^ab^ | 159 (58)^ac^ | 345 (48)^bc^ |  |
| **Laboratory parameters** |  |  |  |  |
| Hematocrit, L/L | 0.38±0.04^ab^ | 0.40±0.06^a^ | 0.40±0.05^b^ | <0.001 |
| Hemoglobin, mmol/L | 7.5±1.0^ab^ | 8.0±1.1^a^ | 8.0±1.2^b^ | <0.001 |
| LDL cholesterol, mmol/L | 2.5 [2.0-3.2] | 2.5 [2.0-3.2] | 2.6 [2.1-3.2] | 0.4 |
| HDL cholesterol, mmol/L | 1.4 [1.1-1.7] | 1.3 [1.1-1.6] | 1.3 [1.1-1.7] | 0.06 |
| TC/HDL cholesterol ratio | 3.0 [2.5-4.1] | 3.3 [2.7-4.2] | 3.2 [2.7-4.1] | 0.2 |
| Triglycerides, mmol/L | 1.53 [1.15-2.23] | 1.71 [1.21-2.28] | 1.73 [1.29-2.35] | 0.05 |
| Total cholesterol, mmol/L | 4.6±1.1 | 4.5±1.2 | 4.7±1.2 | 0.2 |
| HbA1c, mmol/mol | 38 [35-42]^ab^ | 40 [36-45]^ac^ | 42 [37-47]^bc^ | <0.001 |
| Glucose, mmol/L | 5.6 [5.2-6.4] | 5.9 [5.3-7.1] | 6.0 [5.3-7.1] | 0.05 |
| CRP, mg/L | 1.9 [0.6-4.8] | 2.1 [0.8-6.0] | 2.0 [0.8-5.0] | 0.5 |
| eGFR, ml/min/1.73m^2^ | 62±25^ab^ | 53±19^a^ | 52±18^b^ | <0.001 |

ᵃᵇᶜ:Groups with different superscripts differ significantly (p<0.05). Post hoc comparisons: Tukey’s HSD or Games–Howell for normally distributed variables; Dunn’s test (Holm adjustment) for non-normally distributed variables; pairwise comparisons with Bonferroni correction for categorical variables.

Abbreviations: LDL; low-density lipoprotein, HDL; high density lipoprotein, TC; total cholesterol, CRP; C-reactive protein, eGFR; estimated glomerular filtration rate.

Table S5: multinomial logistic regression analysis with hypertension (control) status as dependent variable in the SOT transplantation population. Reference category: recipients without hypertension. Odds ratios for optimal and suboptimal hypertension control are each presented relative to recipients without hypertension. Multivariable logistic regression is adjusted for age, sex and transplantation type.

|  | Optimal control |  |  |  | Suboptimal control | | | |
| --- | --- | --- | --- | --- | --- | --- | --- | --- |
|  | Univariable  OR (95% CI) | P-value | Multivariable  OR(95% CI) | P-value | Univariable  OR (95% CI) | P-value | Multivariable  OR(95% CI) | P-value |
| **Demographics** |  |  |  |  |  |  |  |  |
| Age, years^a^ | 1.01 (1.00-1.03) | 0.1 | 1.01 (0.99–1.03) | 0.2 | 1.03 (1.01-1.04) | <0.001 | 1.03 (1.01–1.04) | 0.001 |
| Sex^b^ |  |  |  |  |  |  |  |  |
| Female | Reference |  | Reference |  | Reference |  | Reference |  |
| Male | 1.95 (1.26-3.03) | 0.003 | 1.74 (1.10–2.76) | 0.02 | 2.21 (1.49-3.29) | <0.001 | 1.98 (1.31–3.01) | 0.001 |
| BMI, kg/m2 | 1.16 (1.09-1.23) | <0.001 | 1.11 (1.05–1.18) | <0.001 | 1.17 (1.11-1.24) | <0.001 | 1.13 (1.07–1.19) | <0.001 |
| Metabolic syndrome, |  |  |  |  |  |  |  |  |
| No | Reference |  | Reference |  | Reference |  | Reference |  |
| Yes | 8.76 (4.15-18.50) | <0.001 | 8.28 (3.84–17.84) | <0.001 | 9.36 (4.62-19.00) | <0.001 | 9.05 (4.38–18.69) | <0.001 |
| Waist circumference, cm | 1.05 (1.02-1.09) | <0.001 | 1.04 (1.01–1.08) | 0.02 | 1.06 (1.03-1.09) | <0.001 | 1.04 (1.01–1.07) | 0.008 |
| Transplantation type^c^ |  |  |  |  |  |  |  |  |
| Kidney | Reference |  | Reference |  | Reference |  | Reference |  |
| Liver | 0.07 (0.03-0.13) | <0.001 | 0.07 (0.03–0.14) | <0.001 | 0.15 (0.09-0.24) | <0.001 | 0.16 (0.09–0.27) | <0.001 |
| Lung | 0.20 (0.11-0.36) | <0.001 | 0.21 (0.12–0.37) | <0.001 | 0.22 (0.14-0.37) | <0.001 | 0.23 (0.14–0.39) | <0.001 |
| Heart | 0.58 (0.15-2.21) | 0.4 | 0.60 (0.15–2.31) | 0.5 | 0.66 (0.19-2.26) | 0.5 | 0.73 (0.21–2.55) | 0.6 |
| **Lifestyle parameters** |  |  |  |  |  |  |  |  |
| Salt intake, g/day | 0.97 (0.90-1.04) | 0.4 | 0.95 (0.88–1.02) | 0.1 | 1.02 (0.96-1.09) | 0.5 | 1.00 (0.93–1.07) | <1.0 |
| Alcohol, units a week |  |  |  |  |  |  |  |  |
| <7 units | Reference |  | Reference |  | Reference |  | Reference |  |
| ≥7 units | 1.58 (0.41-6.12) | 0.5 | 1.25 (0.31–5.06) | 0.8 | 3.54 (1.07-11.77) | 0.04 | 2.92 (0.84–10.11) | 0.09 |
| Smoking |  |  |  |  |  |  |  |  |
| Non-smoker | Reference |  | Reference |  | Reference |  | Reference |  |
| Ex-smoker | 1.32 (0.75-2.33) | 0.3 | 1.20 (0.65–2.20) | 0.6 | 1.36 (0.81-2.28) | 0.3 | 1.17 (0.67–2.03) | 0.6 |
| Current | 2.14 (0.78-5.87) | 0.1 | 1.69 (0.58–4.95) | 0.3 | 1.70 (0.65-4.44) | 0.3 | 1.38 (0.50–3.82) | 0.5 |
| **Medication use** |  |  |  |  |  |  |  |  |
| Immunosuppressives |  |  |  |  |  |  |  |  |
| Tacrolimus | 0.93 (0.38–2.29) | 0.9 | 1.29 (0.49–3.36) | 0.6 | 0.93 (0.41–2.11) | 0.9 | 1.31 (0.55–3.15) | 0.5 |
| Ciclosporin | 1.15 (0.36–3.71) | 0.8 | 0.57 (0.17–1.91) | 0.4 | 1.46 (0.51–4.19) | 0.5 | 0.74 (0.25–2.24) | 0.6 |
| mTOR inhibitors | 0.47 (0.23–1.00) | 0.05 | 0.96 (0.41–2.25) | 0.9 | 0.89 (0.48–1.63) | 0.7 | 1.42 (0.70–2.91) | 0.3 |
| Antiproliferation agents | 1.88 (1.06–3.34) | 0.03 | 1.03 (0.52–2.01) | 0.9 | 1.26 (0.77–2.05) | 0.4 | 0.85 (0.47–1.53) | 0.6 |
| Prednis(ol)on | 6.94 (3.70–13.03) | <0.001 | 2.13 (0.65–6.99) | 0.2 | 3.81 (2.41–6.03) | <0.001 | 1.83 (0.69–4.88) | 0.2 |
| **Medical history** |  |  |  |  |  |  |  |  |
| Cardiovascular event, n(%) | 2.98 (1.58-5.61) | <0.001 | 2.38 (1.15–4.92) | 0.02 | 2.84 (1.56-5.18) | <0.001 | 2.08 (1.04–4.14) | 0.04 |
| Diabetes, n(%) | 1.61 (0.93-2.77) | 0.09 | 1.64 (0.93–2.91) | 0.09 | 2.12 (1.29-3.49) | 0.003 | 1.99 (1.18–3.36) | 0.01 |
| Pre-transplant hypertension, n(%) | 4.74 (2.53–8.86) | <0.001 | 2.82 (1.44–5.51) | 0.002 | 3.51 (1.93–6.38) | <0.001 | 2.02 (1.07–3.84) | 0.03 |
| **Laboratory parameters** |  |  |  |  |  |  |  |  |
| **Blood values** |  |  |  |  |  |  |  |  |
| Hematocrit, % | 1.07 (1.03-1.12) | 0.001 | 1.00 (0.95–1.05) | <1.0 | 1.08 (1.04-1.13) | <0.001 | 1.02 (0.98–1.07) | 0.3 |
| Hemoglobin, mmol/L | 1.39 (1.14–1.69) | 0.001 | 1.05 (0.83–1.32) | 0.7 | 1.46 (1.22–1.75) | <0.001 | 1.15 (0.93–1.42) | 0.2 |
| LDL cholesterol, mmol/L | 1.00 (0.79–1.28) | <1.0 | 1.20 (0.92–1.57) | 0.2 | 1.08 (0.87–1.34) | 0.5 | 1.28 (1.00–1.64) | 0.05 |
| HDL cholesterol, mmol/L | 0.51 (0.31–0.84) | 0.008 | 0.52 (0.30–0.91) | 0.02 | 0.73 (0.47–1.12) | 0.2 | 0.83 (0.51–1.34) | 0.4 |
| Total cholesterol, mmol/L | 0.92 (0.76–1.12) | 0.4 | 1.08 (0.86–1.35) | 0.5 | 1.04 (0.87–1.24) | 0.6 | 1.23 (1.01–1.51) | 0.04 |
| TC/HDL cholesterol ratio | 1.13 (0.93–1.37) | 0.2 | 1.20 (0.98–1.47) | 0.07 | 1.13 (0.94–1.35) | 0.2 | 1.19 (0.99–1.42) | 0.07 |
| Triglycerides, mmol/L | 1.16 (0.89–1.52) | 0.3 | 1.13 (0.87–1.48) | 0.4 | 1.36 (1.06–1.73) | 0.01 | 1.33 (1.04–1.69) | 0.02 |
| HbA1c, mmol/mol | 1.05 (1.02–1.08) | 0.002 | 1.03 (1.00–1.06) | 0.08 | 1.06 (1.04–1.09) | <0.001 | 1.04 (1.01–1.07) | 0.005 |
| Glucose^d^, mmol/L | 1.79 (0.74–4.28) | 0.2 | 2.24 (0.83–6.03) | 0.1 | 2.40 (1.07–5.34) | 0.03 | 2.47 (0.98–6.20) | 0.05 |
| CRP, mg/L | 1.02 (0.99–1.04) | 0.1 | 1.02 (1.00–1.05) | 0.1 | 0.99 (0.97–1.01) | 0.4 | 0.99 (0.97–1.02) | 0.6 |
| eGFR, ml/min/1.73m^2^ | 0.98 (0.97–0.99) | <0.001 | 0.99 (0.98–1.00) | 0.05 | 0.98 (0.97–0.99) | <0.001 | 0.99 (0.97–1.00) | 0.007 |
| Creatinine^d^, µmol/L | 3.83 (1.87–7.85) | <0.001 | 1.90 (0.89–4.02) | 0.1 | 3.97 (2.05–7.69) | <0.001 | 2.07 (1.05–4.08) | 0.04 |

^a^: only adjusted for transplantation type and sex in multivariable regression. ^b^:only adjusted for transplantation type and age in multivariable regression. ^c^: only adjusted for age and sex in multivariable regression. ^d^: logarithmic transformation.

Abbreviations: LDL; low-density lipoprotein, HDL; high density lipoprotein, TC; total cholesterol, CRP; C-reactive protein, eGFR; estimated glomerular filtration rate.

Table S6: Characteristics of the SOT transplantation group with suboptimal hypertension control, stratified by antihypertensive use.

|  | Untreated  n= 146 | Treated  n= 575 | p-value |
| --- | --- | --- | --- |
| **Demographics** |  |  |  |
| Age, years | 55 ±12 | 59 ±13 | 0.001 |
| Sex, n(%) |  |  | 0.03 |
| Male | 81 (56) | 379 (66) |  |
| Female | 65 (44) | 196 (34) |  |
| BMI, kg/m2 | 26.5±4.8 | 27.4±4.1 | 0.03 |
| Metabolic syndrome, n(%) |  |  | 0.09 |
| No | 61 (60) | 195 (50) |  |
| Yes | 40 (40) | 192 (50) |  |
| Waist circumference, cm |  |  |  |
| Male | 100±12 | 101±12 | 0.7 |
| Female | 90±12 | 95±11 | 0.04 |
| Transplantation type, n(%) |  |  |  |
| Kidney | 59 (11) | 474 (89) | <0.001 |
| Liver | 37 (54) | 32 (46) | 0.5 |
| Lung | 44 (47) | 50 (53) | 0.5 |
| Heart | 6 (24) | 19 (76) | 0.009 |
| **Blood pressure parameters** |  |  |  |
| Systolic blood pressure, mmHg | 140±13 | 142±14 | 0.2 |
| Diastolic blood pressure, mmHg | 85±9 | 81±10 | <0.001 |
| Pulse rate, freq/min | 79±14 | 72±12 | <0.001 |
| Pulse pressure, mmHg | 55±14 | 61±17 | <0.001 |
| Mean arterial pressure, mmHg | 103±9 | 101±8 | 0.007 |
| **Life style parameters** |  |  |  |
| Salt intake, g/day | 8.2 [5.5-10.5] | 8.7 [6.5-11.7] | 0.02 |
| Alcohol intake, units a week, n(%) |  |  | 0.4 |
| <7 units | 60 (86) | 202 (81) |  |
| ≥7 units | 10 (14) | 48 (19) |  |
| Smoking, n(%) |  |  | 0.5 |
| Non-smoker | 82 (66) | 261 (61) |  |
| Ex-smoker | 35 (28) | 130 (30) |  |
| Smoker | 8 (6) | 39 (9) |  |
| **Medical history** |  |  |  |
| Cardiovascular event, n(%) | 21 (14) | 171 (30) | <0.001 |
| Diabetes, n(%) | 37 (25) | 195 (34) | 0.06 |
| Pre-transplant hypertension, n(%) | 23 (16) | 200 (35) | <0.001 |
| **Medication** |  |  |  |
| Medication use, n(%) |  |  |  |
| Immunosuppressives |  |  |  |
| Tacrolimus | 141 (97) | 533 (93) | 0.1 |
| Ciclosporin | 3 (2) | 33 (6) | 0.1 |
| mTOR inhibitors | 15 (10) | 64 (11) | 0.9 |
| Antiproliferation agents | 113 (77) | 483 (84) | 0.08 |
| Prednis(ol)on | 113 (77) | 531 (92) | <0.001 |
| **Laboratory parameters** |  |  |  |
| Hematocrit, L/L | 0.38±0.06 | 0.41±0.05 | <0.001 |
| Hemoglobin, mmol/L | 7.7±1.3 | 8.1±1.1 | <0.001 |
| LDL cholesterol, mmol/L | 2.9 [2.3-3.3] | 2.6 [2.0-3.2] | 0.009 |
| HDL cholesterol, mmol/L | 1.4 [1.2-1.8] | 1.3 [1.1-1.7] | 0.08 |
| TC/HDL cholesterol ratio | 3.4 [2.7-3.9] | 3.2 [2.6-4.2] | 0.8 |
| Triglycerides, mmol/L | 1.59 [1.17-2.29] | 1.75 [1.35-2.37] | 0.06 |
| Total cholesterol, mmol/L | 4.8±1.1 | 4.6±1.2 | 0.1 |
| HbA1c, mmol/mol | 39 [35-45] | 42 [37-48] | <0.001 |
| Glucose, mmol/L | 5.9 [5.3-6.8] | 6.0 [5.3-7.3] | 0.2 |
| CRP, mg/L | 1.9 [0.7-4.1] | 2.0 [0.8-5.0] | 0.6 |
| eGFR, ml/min/1.73m^2^ | 57±20 | 51±17 | <0.001 |

Abbreviations: LDL; low-density lipoprotein, HDL; high density lipoprotein, TC; total cholesterol, CRP; C-reactive protein, eGFR; estimated glomerular filtration rate.

Table S7: logistic regression analysis with treatment status (untreated (0) or treated (1)) as dependent variable in the recipients with suboptimal hypertension control. Multivariate logistic regression is adjusted for age, sex and transplantation type.

|  | Univariable  OR (95% CI) | P-value | Multivariable  OR(95% CI) | P-value |
| --- | --- | --- | --- | --- |
| **Demographics** |  |  |  |  |
| Age, years^a^ | 1.02 (1.01-1.04) | <0.001 | 1.03 (1.01–1.05) | <0.001 |
| Sex, n(%)^b^ |  |  |  |  |
| Female | Reference |  | Reference |  |
| Male | 1.55 (1.07-2.24) | 0.02 | 1.55 (1.03–2.34) | 0.04 |
| BMI, kg/m2 | 1.05 (1.01-1.10) | 0.02 | 1.04 (0.99-1.09) | 0.1 |
| Metabolic syndrome, n(%) |  |  |  |  |
| No | Reference |  | Reference |  |
| Yes | 1.50 (0.96-2.36) | 0.07 | 1.58 (0.97–2.60) | 0.07 |
| Waist circumference, cm | 1.03 (1.01–1.06) | 0.01 | 1.02 (0.99–1.04) | 0.2 |
| Transplantation type^c^ |  |  |  |  |
| Kidney | Reference |  | Reference |  |
| Liver | 0.11 (0.06-0.19) | <0.001 | 0.10 (0.06–0.17) | <0.001 |
| Lung | 0.14 (0.09-0.23) | <0.001 | 0.13 (0.08–0.22) | <0.001 |
| Heart | 0.39 (0.16-1.12) | 0.06 | 0.44 (0.18–1.26) | 0.1 |
| **Blood pressure parameters** |  |  |  |  |
| Systolic blood pressure, mmHg | 1.01 (1.00-1.02) | 0.2 | 1.01 (0.99–1.02) | 0.2 |
| Diastolic blood pressure, mmHg | 0.96 (0.94-0.98) | <0.001 | 0.98 (0.96–1.01) | 0.2 |
| Pulse rate, freq/min | 0.96 (0.94-0.97) | <0.001 | 0.98 (0.96-1.00) | 0.08 |
| Pulse pressure, mmHg | 1.02 (1.01-1.03) | <0.001 | 1.02 (1.00–1.03) | 0.05 |
| Mean arterial pressure, mmHg | 0.97 (0.95-0.99) | 0.007 | 1.00 (0.97-1.02) | 0.8 |
| **Lifestyle parameters** |  |  |  |  |
| Salt intake, g/day | 1.07 (1.01-1.13) | 0.03 | 1.07 (1.00-1.14) | 0.04 |
| Alcohol, units per week |  |  |  |  |
| <7 | Reference |  | Reference |  |
| ≥7 | 1.43 (0.70-3.14) | 0.3 | 1.39 (0.61-3.43) | 0.4 |
| Smoking |  |  |  |  |
| Non-smoker | Reference |  | Reference |  |
| Ex-smoker | 1.17 (0.75-1.84) | 0.5 | 0.99 (0.60-1.66) | <1.0 |
| Current | 1.53 (0.72-3.65) | 0.3 | 1.29 (0.55-3.35) | 0.6 |
| **Medical history** |  |  |  |  |
| Cardiovascular event, n(%) | 2.52 (1.57-4.24) | <0.001 | 2.06 (1.14-3.95) | 0.02 |
| Diabetes, n(%) | 1.51 (1.01–2.3) | 0.05 | 1.41 (0.9–2.27) | 0.1 |
| Pre-transplant hypertension, n(%) | 2.85 (1.80–4.70) | <0.001 | 1.67 (0.99–2.88) | 0.06 |
| **Medication** |  |  |  |  |
| Medication use, n(%) |  |  |  |  |
| Immunosuppressives |  |  |  |  |
| Tacrolimus | 0.45 (0.15–1.06) | 0.1 | 0.62 (0.20–1.55) | 0.3 |
| Ciclosporin | 2.90 (1.02–12.19) | 0.08 | 1.60 (0.53–6.93) | 0.5 |
| mTOR inhibitors | 1.09 (0.62–2.05) | 0.8 | 1.35 (0.67–2.89) | 0.4 |
| Antiproliferation agents | 1.53 (0.97–2.38) | 0.06 | 1.27 (0.71–2.21) | 0.4 |
| Prednis(ol)on | 3.52 (2.14–5.77) | <0.001 | 0.60 (0.20–1.64) | 0.3 |
| **Laboratory parameters** |  |  |  |  |
| **Blood values** |  |  |  |  |
| Hematocrit, % | 1.08 (1.04–1.12) | <0.001 | 1.01 (0.97–1.05) | 0.6 |
| Hemoglobin, mmol/L | 1.34 (1.14-1.58) | <0.001 | 1.04 (0.86-1.26) | 0.7 |
| LDL cholesterol, mmol/L | 0.84 (0.69-1.03) | 0.09 | 1.01 (0.81–1.27) | 0.9 |
| HDL cholesterol, mmol/L | 0.65 (0.43-0.98) | 0.04 | 0.65 (0.40-1.07) | 0.09 |
| Total cholesterol, mmol/L | 0.88 (0.75-1.04) | 0.1 | 1.05 (0.87-1.27) | 0.6 |
| TC/HDL cholesterol ratio | 1.09 (0.94-1.29) | 0.3 | 1.23 (1.03-1.47) | 0.02 |
| Triglycerides, mmol/L | 1.20 (1.00–1.47) | 0.07 | 1.25 (1.03–1.57) | 0.03 |
| HbA1c, mmol/mol | 1.04 (1.01–1.06) | 0.001 | 1.02 (1.00-1.04) | 0.08 |
| Glucose, mmol/L | 1.12 (1.02-1.24) | 0.02 | 1.16 (1.04-1.31) | 0.01 |
| CRP, mg/L | 1.00 (0.97-1.03) | 0.8 | 1.01 (0.98–1.04) | 0.6 |
| eGFR, ml/min/1.73m^2^ | 0.98 (0.97-0.99) | <0.001 | 0.99 (0.97–1.00) | 0.01 |
| Creatinine ^d^, µmol/L | 2.75 (1.48–5.21) | 0.002 | 2.27 (1.14–4.65) | 0.02 |

^a^: only adjusted for transplantation type and sex in multivariable regression. ^b^:only adjusted for transplantation type and age in multivariable regression. ^c^: only adjusted for age and sex in multivariable regression. ^d^: logarithmic transformation.

Abbreviations: LDL; low-density lipoprotein, HDL; high density lipoprotein, TC; total cholesterol, CRP; C-reactive protein, eGFR; estimated glomerular filtration rate.

Table S8: Characteristics of kidney transplant recipients with suboptimal hypertension control, stratified by use of antihypertensives.

|  | Untreated  n= 59 | Treated  n= 474 | P-value |
| --- | --- | --- | --- |
| **Demographics** |  |  |  |
| Age, years | 53±14 | 59±14 | 0.001 |
| Sex, n(%) |  |  | 0.006 |
| Male | 28 (48) | 316 (67) |  |
| Female | 31 (52) | 158 (33) |  |
| BMI, kg/m2 | 27.2±5.1 | 27.5±4.0 | 0.6 |
| Metabolic syndrome, n(%) |  |  | <1.0 |
| No | 24 (51) | 162 (50) |  |
| Yes | 23 (49) | 159 (50) |  |
| Waist circumference, cm |  |  |  |
| Male | 97±8 | 101±12 | 0.3 |
| Female | 94±11 | 96±11 | 0.6 |
| **Blood pressure parameters** |  |  |  |
| Systolic blood pressure, mmHg | 139±12 | 141±13 | 0.1 |
| Diastolic blood pressure, mmHg | 83±8 | 80±10 | 0.03 |
| Pulse rate, freq/min | 75±12 | 70±12 | 0.005 |
| Pulse pressure, mmHg | 55±13 | 61±17 | 0.01 |
| Mean arterial pressure, mmHg | 102±7 | 101±8 | 0.4 |
| **Life style parameters** |  |  |  |
| Salt intake, g/day | 8.1 [5.3-10.5] | 8.7 [6.5-11.7] | 0.07 |
| Alcohol units a week, n(%) |  |  | 0.6 |
| <7 units | 24 (86) | 164 (80) |  |
| ≥7 units | 4 (14) | 41 (20) |  |
| Smoking, n(%) |  |  | 0.8 |
| Non-smoker | 27 (56) | 202 (59) |  |
| Ex-smoker | 17 (36) | 104 (31) |  |
| Smoker | 4 (8) | 35 (10) |  |
| **Medical history** |  |  |  |
| Cardiovascular event, n(%) | 8 (14) | 139 (29) | 0.02 |
| Diabetes, n(%) | 11 (19) | 165 (35) | 0.02 |
| Pre-transplant hypertension, n(%) | 20 (34) | 184 (39) | 0.6 |
| **Medication** |  |  |  |
| Medication use, n(%) |  |  |  |
| Immunosuppressives |  |  |  |
| Tacrolimus | 56 (95) | 438 (92) | 0.7 |
| Ciclosporin | 2 (3) | 30 (6) | 0.5 |
| mTOR inhibitors | 2 (3) | 53 (11) | 0.1 |
| Antiproliferation agents | 54 (92) | 399 (84) | 0.2 |
| Prednis(ol)on | 58 (98) | 470 (99) | <1.0 |
| **Medication use** |  |  |  |
| Number of antihypertensives, n(%) |  |  | <0.001 |
| 0 | 59 (100) | 0 (0) |  |
| 1 | 0 (0) | 158 (33) |  |
| >1 | 0 (0) | 316 (67) |  |
| **Laboratory parameters** |  |  |  |
| Hematocrit, L/L | 0.41±0.06 | 0.41±0.05 | 0.3 |
| Hemoglobin, mmol/L | 8.1±1.2 | 8.3±1.1 | 0.4 |
| LDL cholesterol, mmol/L | 2.8 [2.1-3.3] | 2.5 [2.0-3.1] | 0.1 |
| HDL cholesterol, mmol/L | 1.4 [1.2-1.8] | 1.3 [1.1-1.6] | 0.06 |
| TC/HDL cholesterol ratio | 3.2 [2.7-3.8] | 3.2 [2.6-4.1] | 0.5 |
| Triglycerides, mmol/L | 1.53 [1.17-2.28] | 1.73 [1.37-2.37] | 0.1 |
| Total cholesterol, mmol/L | 4.7±1.1 | 4.5±1.1 | 0.2 |
| HbA1c, mmol/mol | 40 [35-46] | 42 [38-48] | 0.03 |
| Glucose, mmol/L | 5.6 [5.1-6.4] | 5.9 [5.2-7.2] | 0.07 |
| CRP, mg/L | 1.7 [0.6-3.6] | 1.9 [0.8-4.7] | 0.4 |
| eGFR, ml/min/1.73m^2^ | 54±19 | 51±17 | 0.3 |

Abbreviations: LDL; low-density lipoprotein, HDL; high density lipoprotein, TC; total cholesterol, CRP; C-reactive protein, eGFR; estimated glomerular filtration rate.

Table S9: Characteristics of liver transplant recipients with suboptimal hypertension control, stratified by antihypertensive use.

|  | Untreated  n= 37 | Treated  n= 32 | P-value |
| --- | --- | --- | --- |
| **Demographics** |  |  |  |
| Age, years | 57±9 | 58±7 | 0.6 |
| Sex, n(%) |  |  | 0.9 |
| Male | 25 (68) | 20 (63) |  |
| Female | 12 (32) | 12 (38) |  |
| BMI, kg/m2 | 26.6±5.2 | 27.8±4.6 | 0.3 |
| Metabolic syndrome, n(%) |  |  | <0.001 |
| No | 18 (78) | 4 (17) |  |
| Yes | 5 (22) | 19 (83) |  |
| Waist circumference, cm |  |  |  |
| Male | 95±12 | 110±9 | 0.01 |
| Female | 87±18 | 95±10 | 0.5 |
| **Blood pressure parameters** |  |  |  |
| Systolic blood pressure, mmHg | 141±15 | 144±13 | 0.3 |
| Diastolic blood pressure, mmHg | 85± 10 | 84± 12 | 0.7 |
| Pulse rate, freq/min | 76±10 | 78±13 | 0.5 |
| Pulse pressure, mmHg | 55±13 | 60±19 | 0.2 |
| Mean arterial pressure, mmHg | 104±10 | 104±9 | 0.8 |
| **Life style parameters** |  |  |  |
| Salt intake, g/day | 8.1 [5.7-10.3] | 9.3 [8.9-14.1] | 0.03 |
| Alcohol intake a week, n(%) |  |  |  |
| <7 units | 12 (100) | 16 (100) | NA |
| ≥7 units | 0 (0) | 0 (0) |  |
| Smoking, n(%) |  |  | 0.6 |
| Non-smoker | 19 (61) | 12 (48) |  |
| Ex-smoker | 9 (29) | 9 (36) |  |
| Smoker | 3 (10) | 4 (16) |  |
| **Medical history** |  |  |  |
| Cardiovascular event, n(%) | 2 (5) | 5 (16) | 0.3 |
| Diabetes, n(%) | 12 (32) | 16 (50) | 0.2 |
| Pre-transplant hypertension, n(%) | 2 (5) | 11 (34) | 0.006 |
| **Medication** |  |  |  |
| Medication use, n(%) |  |  |  |
| Immunosuppressives |  |  |  |
| Tacrolimus | 36 (97) | 29 (91) | 0.5 |
| Ciclosporin | 0 (0) | 0 (0) | NA |
| mTOR inhibitors | 10 (27) | 11 (34) | 0.7 |
| Antiproliferation agents | 15 (41) | 18 (56) | 0.3 |
| Prednis(ol)on | 8 (22) | 3 (9) | 0.3 |
| **Medication use** |  |  |  |
| Number of antihypertensives, n(%) |  |  | <0.001 |
| 0 | 37 (100) | 0 (0) |  |
| 1 | 0 (0.0) | 23 (72) |  |
| >1 | 0 (0.0) | 9 (28) |  |
| **Laboratory parameters** |  |  |  |
| Hematocrit, L/L | 0.38±0.07 | 0.38±0.06 | 0.8 |
| Hemoglobin, mmol/L | 7.8±1.4 | 7.8±1.3 | 0.9 |
| LDL cholesterol, mmol/L | 2.7 [2.4-3.2] | 2.8 [2.1-3.4] | <1.0 |
| HDL cholesterol, mmol/L | 1.3 [1.0-1.5] | 1.0 [0.9-1.3] | 0.06 |
| TC/HDL cholesterol ratio | 3.4 [2.9-3.9] | 4.3 [3.2-5.4] | 0.01 |
| Triglycerides, mmol/L | 1.29 [0.99-1.91] | 1.81 [1.29-2.82] | 0.02 |
| Total cholesterol, mmol/L | 4.4±1.0 | 4.8±1.5 | 0.2 |
| HbA1c, mmol/mol | 37 [33-42] | 40 [36-55] | 0.06 |
| Glucose, mmol/L | 5.7 [5.3-6.9] | 6.7 [5.7-8.9] | 0.04 |
| CRP, mg/L | 2.4 [1.3-4.1] | 2.9 [1.5-6.5] | 0.3 |
| eGFR, ml/min/1.73m^2^ | 68±19 | 57±24 | 0.06 |

Abbreviations: LDL; low-density lipoprotein, HDL; high density lipoprotein, TC; total cholesterol, CRP; C-reactive protein, eGFR; estimated glomerular filtration rate.

Table S10: Characteristics of lung transplant recipients with suboptimal hypertension control, stratified by antihypertensive use.

|  | Untreated  n = 44 | Treated  n = 50 | P-value |
| --- | --- | --- | --- |
| **Demographics** |  |  |  |
| Age, years | 56±10 | 60±6 | 0.04 |
| Sex, n(%) |  |  | 0.6 |
| Male | 24 (55) | 31 (62) |  |
| Female | 20 (46) | 19 (38) |  |
| BMI, kg/m2 | 25.7±4.2 | 26.5±3.4 | 0.3 |
| Metabolic syndrome, n(%) |  |  | 0.8 |
| No | 17 (59) | 20 (64) |  |
| Yes | 12 (41) | 11 (36) |  |
| Waist circumference, cm |  |  |  |
| Male | 107±15 | 97±10 | 0.05 |
| Female | 87±11 | 92±12 | 0.4 |
| **Blood pressure parameters** |  |  |  |
| Systolic blood pressure, mmHg | 143±14 | 144±17 | 0.9 |
| Diastolic blood pressure, mmHg | 87±10 | 86±8 | 0.7 |
| Pulse rate, freq/min | 88±15 | 81±14 | 0.1 |
| Pulse pressure, mmHg | 57±16 | 58±15 | 0.7 |
| Mean arterial pressure, mmHg | 105±9 | 105±10 | 0.9 |
| **Life style parameters** |  |  |  |
| Salt intake, g/day | 8.2 [5.7-10.5] | 7.8 [6.2-11.1] | 0.9 |
| Alcohol intake a week, n(%) |  |  | 0.8 |
| <7 units | 19 (76) | 15 (68) |  |
| ≥7 units | 6 (24) | 7 (32) |  |
| Smoking, n(%) |  |  | 0.3 |
| Non-smoker | 30 (75) | 31 (66) |  |
| Ex-smoker | 9 (23) | 16 (34) |  |
| Smoker | 1 (2) | 0 (0) |  |
| **Medical history** |  |  |  |
| Cardiovascular event, n(%) | 5 (11) | 8 (16) | 0.7 |
| Diabetes, n(%) | 10 (23) | 11 (22) | <1.0 |
| Pre-transplant hypertension, n(%) | 1 (2) | 4 (8) | 0.4 |
| **Medication** |  |  |  |
| Medication use, n(%) |  |  |  |
| Immunosuppressives |  |  |  |
| Tacrolimus | 44 (100) | 50 (100) | NA |
| Ciclosporin | 0 (0) | 0 (0) | NA |
| mTOR inhibitors | 3 (7) | 0 (0) | 0.2 |
| Antiproliferation agents | 38 (86) | 47 (94) | 0.4 |
| Prednis(ol)on | 44 (100) | 50 (100) | NA |
| **Medication use** |  |  |  |
| Number of antihypertensives, n(%) |  |  | <0.001 |
| 0 | 44 (100) | 0 (0) |  |
| 1 | 0 (0) | 38 (76) |  |
| >1 | 0 (0) | 12 (24) |  |
| **Laboratory parameters** |  |  |  |
| Hematocrit, L/L | 0.36 (±0.04) | 0.36 (±0.04) | 0.8 |
| Hemoglobin, mmol/L | 7.2 (±1.0) | 7.3 (±0.9) | 0.7 |
| LDL cholesterol, mmol/L | 3.1 [2.5-3.6] | 3.1 [2.5-3.6] | 0.7 |
| HDL cholesterol, mmol/L | 1.5 [1.3-2.0] | 1.6 [1.3-1.8] | 0.7 |
| TC/HDL cholesterol ratio | 3.4 [2.7-3.9] | 3.2 [2.7-4.1] | <1.0 |
| Triglycerides, mmol/L | 2.02 [1.40-2.66] | 1.82 [1.35-2.37] | 0.4 |
| Total cholesterol, mmol/L | 5.3±1.1 | 5.4±1.3 | 0.6 |
| HbA1c, mmol/mol | 40 [36-45] | 42 [38-47] | 0.3 |
| Glucose, mmol/L | 6.1 [5.7-6.9] | 6.7 [5.8-7.4] | 0.1 |
| CRP, mg/L | 1.8 [0.7-4.3] | 3.2 [1.2-7.0] | 0.1 |
| eGFR, ml/min/1.73m^2^ | 53±21 | 45±18 | 0.05 |

Abbreviations: LDL; low-density lipoprotein, HDL; high density lipoprotein, TC; total cholesterol, CRP; C-reactive protein, eGFR; estimated glomerular filtration rate.

Table S11: Characteristics of heart transplant recipients with suboptimal hypertension control, stratified by antihypertensive use.

|  | Untreated  n= 6 | Treated  n= 19 | p-value |
| --- | --- | --- | --- |
| **Demographics** |  |  |  |
| Age, years | 54±14 | 52±9 | 0.7 |
| Sex, n(%) |  |  | <1.0 |
| Male | 4 (67) | 12 (63) |  |
| Female | 2 (33) | 7 (37) |  |
| BMI, kg/m2 | 23.5±1.8 | 25.5±5.2 | 0.4 |
| Metabolic syndrome, n(%) |  |  | <1.0 |
| No | 2 (100) | 9 (75) |  |
| Yes | 0 (0) | 3 (25) |  |
| **Blood pressure parameters** |  |  |  |
| Systolic blood pressure, mmHg | 132±11 | 142±16 | 0.2 |
| Diastolic blood pressure, mmHg | 90±8 | 86±8 | 0.2 |
| Pulse rate, freq/min | 87±13 | 89±9 | 0.7 |
| Pulse pressure, mmHg | 42±15 | 56±14 | 0.04 |
| Mean arterial pressure, mmHg | 104±5 | 104±9 | <1.0 |
| **Life style parameters** |  |  |  |
| Salt intake, g/day | NA [NA-NA] | 4.2 [4.2-4.2] | NA |
| Alcohol intake a week, n(%) |  |  |  |
| <7 units | 5 (100) | 7 (100) | NA |
| >7 units | 0 (0) | 0 (0) |  |
| Smoking, n(%) |  |  | <1.0 |
| Non-smoker | 6 (100) | 16 (94) |  |
| Ex-smoker | 0 (0) | 1 (6) |  |
| Smoker | 0 (0) | 0 (0) |  |
| **Medical history** |  |  |  |
| Cardiovascular event, n(%) | 6 (100) | 19 (100) | NA |
| Diabetes, n(%) | 4 (67) | 3 (16) | 0.06 |
| Pre-transplant hypertension, n(%) | 0 (0) | 1 (5) | <1.0 |
| **Medication** |  |  |  |
| Medication use, n(%) |  |  |  |
| Immunosuppressives |  |  |  |
| Tacrolimus | 5 (83) | 16 (84) | <1.0 |
| Ciclosporin | 1 (17) | 3 (16) | <1.0 |
| mTOR inhibitors | 0 (0) | 0 (0) | NA |
| Antiproliferation agents | 6 (100) | 19 (100) | NA |
| Prednis(ol)on | 3 (50) | 8 (42) | <1.0 |
| **Medication use** |  |  |  |
| Number of antihypertensives, n(%) |  |  | <0.001 |
| 0 | 6 (100) | 0 (0) |  |
| 1 | 0 (0.0) | 11 (58) |  |
| >1 | 0 (0.0) | 8 (42) |  |
| **Laboratory parameters** |  |  |  |
| Hematocrit, L/L | 0.36±0.02 | 0.36±0.04 | <1.0 |
| Hemoglobin, mmol/L | 7.1±0.3 | 7.1±0.8 | <1.0 |
| LDL cholesterol, mmol/L | 2.6 [2.4-3.0] | 2.4 [2.0-3.2] | 0.9 |
| HDL cholesterol, mmol/L | 1.2 [1.1-1.4] | 1.2 [1.1-1.6] | 0.7 |
| TC/HDL cholesterol ratio | 3.5 [3.2-3.7] | 3.1 [2.4-3.9] | 0.4 |
| Triglycerides, mmol/L | 1.41 [1.14-1.72] | 1.52 [1.06-1.84] | 0.8 |
| Total cholesterol, mmol/L | 4.2±0.8 | 4.3±1.0 | 0.9 |
| HbA1c, mmol/mol | 38 [32-41] | 37 [34-39] | 0.9 |
| Glucose, mmol/L | 5.7 [5.5-6.4] | 5.8 [5.4-6.6] | 0.7 |
| CRP, mg/L | 6.0 [1.6-11.0] | 1.1 [0.6-5.0] | 0.2 |
| eGFR, ml/min/1.73m^2^ | 69±21 | 60±18 | 0.3 |

Abbreviations: LDL; low-density lipoprotein, HDL; high density lipoprotein, TC; total cholesterol, CRP; C-reactive protein, eGFR; estimated glomerular filtration rate.

Figure S2: Distribution of number of antihypertensives across SOT recipients stratified according to the threshold of >135/>85 mmHg

Figure S3: Distribution of number of antihypertensives across heart, liver, lung and kidney transplantation recipients stratified according to the threshold of >135/>85 mmHg

Figure S4: Distribution of number of antihypertensives across heart, liver, lung and kidney transplantation recipients stratified according to the threshold of >140/>90 mmHg

Supplementary Table S12: Sensitivity analysis of hypertension prevalence and suboptimal hypertension control according to blood pressure measurement strategy.

| **Population** | **Total SOTR, n** | **Hypertension, n(%)** | **Suboptimal control, n(%)** |
| --- | --- | --- | --- |
| Primary analysis | 1112 | 997 (90) | 721 (72) |
| Protocolized study visits only | 433 | 386 (89) | 298 (77) |

Abbreviation: SOTR; solid organ transplant recipients.

Table S13: Multinomial logistic regression analysis with hypertension (control) status as dependent variable in the SOT transplantation population. Reference category: recipients without hypertension. Odds ratios for optimal and suboptimal hypertension control are each presented relative to recipients without hypertension. Multivariable logistic regression is adjusted for age, sex and transplantation type.

|  | Optimal control |  |  |  | Suboptimal control | | | |
| --- | --- | --- | --- | --- | --- | --- | --- | --- |
|  | Univariable  OR (95% CI) | P-value | Multivariable  OR(95% CI) | P-value | Univariable  OR (95% CI) | P-value | Multivariable  OR(95% CI) | P-value |
| **Medical history** |  |  |  |  |  |  |  |  |
| Cardiovascular event, n(%) | 2.98 (1.58-5.61) | <0.001 | 2.38 (1.15–4.92) | 0.02 | 2.84 (1.56-5.18) | <0.001 | 2.08 (1.04–4.14) | 0.04 |
| Myocardial infarction | 3.23 (0.73–14.34) | 0.1 | 1.81 (0.39-8.43) | 0.5 | 3.84 (0.92–15.99) | 0.06 | 1.95 (0.45-8.48) | 0.4 |
| Heart failure | 2.68 (1.01–7.08) | 0.05 | 3.43 (0.86-13.80) | 0.08 | 2.25 (0.89–5.71) | 0.09 | 2.62 (0.68-10.10) | 0.2 |
| Peripheral vascular disease | 2.60 (0.75–8.98) | 0.1 | 1.69 (0.47-6.07) | 0.4 | 3.69 (1.14–11.92) | 0.03 | 2.27 (0.68-7.60) | 0.2 |
| CVA/TIA | 2.78 (1.05–7.35) | 0.04 | 2.14 (0.77-5.90) | 0.1 | 1.89 (0.74–4.82) | 0.2 | 1.35 (0.51-3.60) | 0.5 |

Abbreviations: CVA; cerebrovascular accident, TIA; transient ischemic attack.

The cardiovascular event categories comprised 63 myocardial infarctions, 102 cases of heart failure, 86 cases of peripheral vascular disease, and 93 CVA/TIA events.

Table S14: Logistic regression analysis with treatment status (untreated (0) or treated (1)) as dependent variable in the recipients with suboptimal hypertension control. Multivariate logistic regression is adjusted for age, sex and transplantation type.

|  | Univariable  OR (95% CI) | P-value | Multivariable  OR(95% CI) | P-value |
| --- | --- | --- | --- | --- |
| **Medical history** |  |  |  |  |
| Cardiovascular event, n(%) | 2.52 (1.57-4.24) | <0.001 | 2.06 (1.14-3.95) | 0.02 |
| Myocardial infarction | 3.85 (1.38–16.06) | 0.03 | 1.94 (0.65–8.38) | 0.3 |
| Heart failure | 1.71 (0.87–3.77) | 0.1 | 3.02 (0.98–13.37) | 0.09 |
| Peripheral vascular disease | 1.64 (0.83–3.63) | 0.2 | 0.96 (0.45–2.28) | 0.9 |
| CVA/TIA | 7.62 (2.33–46.85) | 0.005 | 7.06 (1.97–45.54) | 0.01 |

Abbreviations: CVA; cerebrovascular accident, TIA; transient ischemic attack.

The cardiovascular event categories comprised 63 myocardial infarctions, 102 cases of heart failure, 86 cases of peripheral vascular disease, and 93 CVA/TIA events.

Table S15: Prescribed ACEi, ARB, and/or beta-blocker and corresponding metabolite detection.

| **Prescribed medication** | **Samples (n)** | **Metabolite detected, n (%)** |
| --- | --- | --- |
| No prescribed ACEi, ARB or beta-blocker | 60 | 0 (0) |
| ACEi |  |  |
| Enalapril | 9 | 8 (89) |
| Lisinopril | 6 | 6 (100) |
| Perindopril | 3 | 3 (100) |
| Quinapril | 0 | 0 (100) |
| Captopril | 0 | 0 (100) |
| Ramipril | 0 | 0 (100) |
| ARB |  |  |
| Candesartan | 0 | 0 (100) |
| Irbesartan | 9 | 7 (78) |
| Losartan | 4 | 4 (100) |
| Telmisartan | 0 | 0 (100) |
| Valsartan | 0 | 0 (100) |
| Beta-blocker |  |  |
| Atenolol | 3 | 2 (67) |
| Bisoprolol | 11 | 11 (100) |
| Carvedilol | 0 | 0 (100) |
| Labetalol | 0 | 0 (100) |
| Metoprolol | 71 | 69 (97) |
| Sotalol | 1 | 1 (100) |
| Nebivolol | 2 | 0 (0) |
| Propranolol | 1 | 1 (100) |

Abbreviations: ACEi; angiotensin-converting enzyme-inhibitors, ARB; angiotensin receptor blockers. Metabolite detection indicates recent medication exposure but does not allow assessment of adherence to the prescribed dose or dosing schedule.
